# Efficacy of Human Recombinant Growth Hormone in Females of a Non-Obese Hyperglycemic Mouse Model after Birth with Low Birth Weight

**DOI:** 10.3390/ijms25126294

**Published:** 2024-06-07

**Authors:** Wataru Tokunaga, Nobuhiko Nagano, Kengo Matsuda, Kimitaka Nakazaki, Shoichi Shimizu, Koh Okuda, Ryoji Aoki, Kazumasa Fuwa, Hitohiko Murakami, Ichiro Morioka

**Affiliations:** 1 Department of Pediatrics and Child Health, Nihon University School of Medicine, Tokyo 173-8610, Japan; tokunaga.wataru@nihon-u.ac.jp (W.T.); matsuda.kengo@nihon-u.ac.jp (K.M.); nakazaki.kimitaka@nihon-u.ac.jp (K.N.); shimizu.shoichi@nihon-u.ac.jp (S.S.); kohokuda615@gmail.com (K.O.); aoki.ryoji@nihon-u.ac.jp (R.A.); fuwa.kazumasa@nihon-u.ac.jp (K.F.); morioka.ichiro@nihon-u.ac.jp (I.M.); 2 Jin Children’s Clinic, Tokyo 145-0065, Japan; jin.sinn.md@gmail.com

**Keywords:** developmental origins of health and disease, low birth weight, growth hormone, insulin resistance, oxidative stress, mitochondrial dysfunction, muscle fiber type

## Abstract

We examined whether the administration of growth hormone (GH) improves insulin resistance in females of a non-obese hyperglycemic mouse model after birth with low birth weight (LBW), given that GH is known to increase muscle mass. The intrauterine Ischemia group underwent uterine artery occlusion for 15 min on day 16.5 of gestation. At 4 weeks of age, female mice in the Ischemia group were divided into the GH-treated (Ischemia-GH) and non-GH-treated (Ischemia) groups. At 8 weeks of age, the glucose metabolism, muscle pathology, and metabolome of liver were assessed. The insulin resistance index improved in the Ischemia-GH group compared with the Ischemia group (*p* = 0.034). The percentage of type 1 muscle fibers was higher in the Ischemia-GH group than the Ischemia group (*p* < 0.001); the muscle fiber type was altered by GH. In the liver, oxidative stress factors were reduced, and ATP production was increased in the Ischemia-GH group compared to the Ischemia group (*p* = 0.014), indicating the improved mitochondrial function of liver. GH administration is effective in improving insulin resistance by increasing the content of type 1 muscle fibers and improving mitochondrial function of liver in our non-obese hyperglycemic mouse model after birth with LBW.

## 1. Introduction

Low birth weight (LBW) infants have recently been identified as a risk factor for developing lifestyle-related diseases, such as type 2 diabetes mellitus (T2DM) and cardiovascular disease later in life [1,2]. Further epidemiological studies suggest that not only lifestyle-related diseases but also the risks of chronic diseases, such as neurological disorders, are increased [3]. Factors contributing to LBW can be broadly classified into fetal factors, maternal factors, and placental factors. Exposure to low nutrition in utero due to maternal stress or placental insufficiency is a cause of LBW. Metabolic changes occur in the fetus to store energy efficiently and adapt to the low-nutrient environment. In stark contrast, the infant is exposed to overnutrition after birth, increasing susceptibility to lifestyle-related diseases [4,5]. This concept is now known as the developmental origins of health and disease (DOHaD) theory [6]. In Japan, while the number of births is decreasing, the proportion of LBW infants is not [7]. Therefore, reducing the risk of future lifestyle-related diseases is an important issue in terms of medical resources and socioeconomics.

According to the DOHaD theory, LBW infants are more likely to develop obese-related T2DM in the future [2,6]. However, some patients develop non-obese T2DM at a young age [8,9]. For example, Urakami et al. reported that 11% of patients diagnosed with T2DM at a young age were non-obese and were characterized by a high proportion of females (83%) and LBW infants (20%) [10]. Moreover, no significant difference has been reported in the mean body mass index (BMI) of diabetic and non-diabetic patients in Japan [11]. This suggests a certain number of patients with non-obese T2DM within the diabetes population.

We selected the non-obese hyperglycemic mouse model after birth with LBW as a model for non-obese T2DM [12]. Female mice from this model were chosen referencing the findings of Urakami [10]. In this model, Katayama et al. reported that mitochondrial dysfunction induced by oxidative stress reduces lean body mass and triggers myogenic insulin resistance [12]. We have decided to administer growth hormone (GH) to the non-obese hyperglycemic mouse model after birth with LBW to prevent the reduction in lean body mass.

GH promotes muscle growth via insulin-like growth factor 1 (IGF-1) and triglyceride breakdown in adipose tissue, contributing to improve body composition [13,14]. In fact, GH has been applied to treat Prader–Willi syndrome to improve patient body composition [15,16]. Meanwhile, GH deficiency causes a decrease in lean body mass, including muscle and bone mass, and an increase in visceral fat [17]. Although GH elevates blood glucose, its long-term use improves body composition without exacerbating insulin resistance [13,14]. However, it is not clear whether GH administration is effective against non-obese T2DM.

In this study, anticipating the effects of GH previously described, we sought to prevent the loss of lean body mass and improve body composition and insulin resistance in a non-obese hyperglycemic mouse model after birth with LBW by administering GH.

## 2. Results

### 2.1. Body Weight and Body Composition

**Figure 1:**
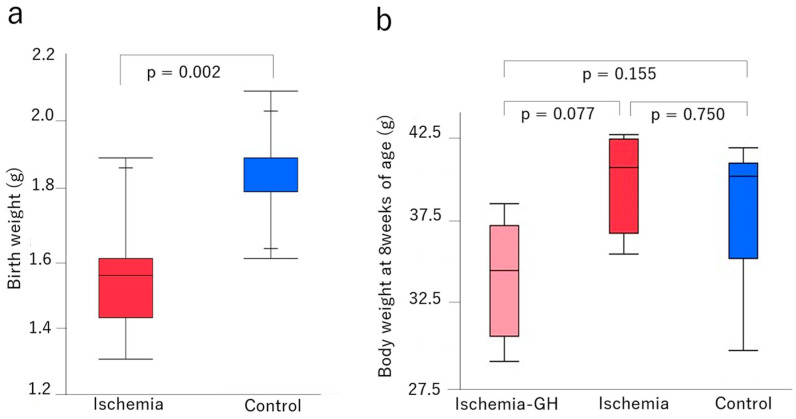
Body weight [median (min, max), g]. (**a**) Birthweight on the first day after birth (Ischemia: *n* = 12, Control: *n* = 6) [Ischemia: 1.55 (1.3, 1.9); Control: 1.9 (1.8, 2.1)]. (**b**) Body weight at 8 weeks of age (*n* = 6/group) [Ischemia-GH: 34.4 (29.2, 38.2); Ischemia: 40.2 (35.3, 42.1); Control 39.7 (29.8, 41.4)].

**Figure 2:**
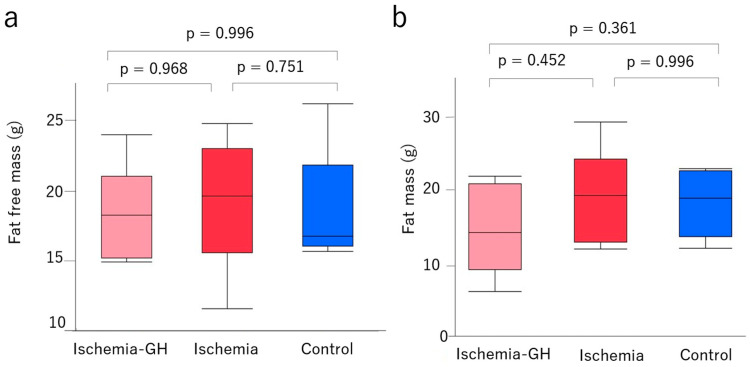
Body composition (*n* = 6/group) [median (min, max), g]. (**a**) Fat-free mass [Ischemia-GH: 18.29 (14.96, 24.09); Ischemia: 19.69 (11.59, 24.87); Control: 16.83 (15.72, 26.33)]. (**b**) Fat mass [Ischemia-GH: 15.09 (7.00, 22.88); Ischemia: 25.23 (12.82, 30.37); Control: 19.85 (12.95, 23.95)].

The birth weight was significantly lower in the Ischemia group [1.5 g (1.3, 1.9) vs. 1.9 g (1.8, 2.1), *p* = 0.002] than in the Control group (Figure 1a). This indicates that the ischemic manipulation was adequate. A body weight at 8 weeks of age did not differ significantly among all groups (Figure 1b). Lean and fat masses also did not differ among groups at 8 weeks of age (Figure 2).

### 2.2. Glucose Metabolism and Serum Lipoproteins

**Figure 3:**
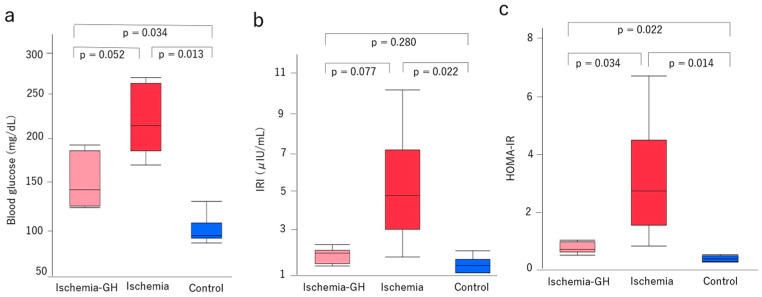
Glucose metabolism markers (*n* = 6/group) [median (min, max)]. (**a**) Fasting blood glucose [Ischemia-GH: 148 (129, 198); Ischemia: 219 (176, 272); Control: 98 (90, 136), mg/dL]. (**b**) Serum immunoreactive insulin [Ischemia-GH: 2.06 (1.43, 2.48); Ischemia: 4.86 (1.89, 10.01); Control: 1.45 (1.08, 2.17), µIU/mL]. (**c**) Homeostasis model assessment of insulin resistance (HOMA-IR) [Ischemia-GH: 0.70 (0.50, 1.02), Ischemia: 2.72 (0.82, 6.72); Control: 0.37 (0.26, 0.52)].

Fasting blood glucose levels at 8 weeks of age showed no significant differences between the Ischemia-GH and Ischemia groups [148 mg/dL (129, 198) vs. 219 mg/dL (176, 272), *p* = 0.052] (Figure 3a). However, the fasting blood glucose of the Control group was significantly lower than the Ischemia and Ischemia-GH groups [Control: 98 mg/dL (90, 136) vs. Ischemia-GH: 148 mg/dL (129, 198), *p* = 0.034] [Control: 98 mg/dL (90, 136) vs. Ischemia: 219 mg/dL (176, 272), *p* = 0.013]. 

Serum immunoreactive insulin (IRI) at 8 weeks of age did not differ significantly between the Ischemia-GH and Ischemia groups [2.06 μIU/mL (1.43, 2.48) vs. 4.86 μIU/mL (1.89, 10.01), *p* = 0.077], but they tended to be higher in the Ischemia group. Moreover, the IRI was significantly higher in the Ischemia group than in the Control group [4.86 μIU/mL (1.89, 10.01) vs. 1.45 μIU/mL (1.08, 2.17), *p* = 0.022] (Figure 3b). 

The homeostatic model assessment of insulin resistance (HOMA-IR) at 8 weeks of age was significantly higher in the Ischemia group than in the other two groups [Ischemia: 2.72 (0.82, 6.72) vs. Ischemia-GH: 0.70 (0.50, 1.02), *p* = 0.034] [Ischemia: 2.72 (0.82, 6.72) vs. Control: 0.37(0.26, 0.52), *p* = 0.014] (Figure 3c).

**Figure 4:**
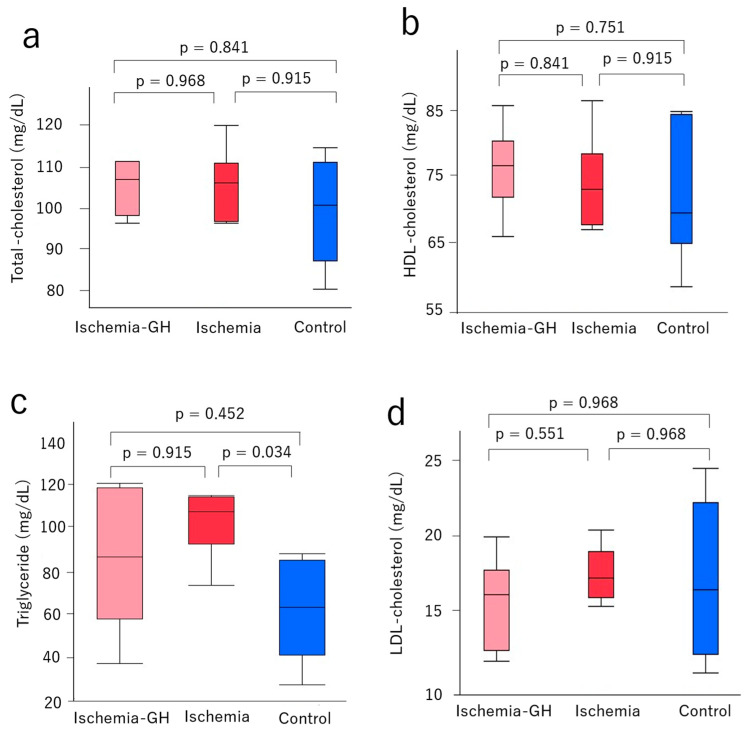
Serum lipoprotein levels (*n* = 6/group) [median (min, max), mg/dL]. (**a**) Total cholesterol [Ischemia-GH: 106 (96, 111); Ischemia: 106 (96, 120); Control: 100 (80, 114)]. (**b**) High-density lipoprotein cholesterol [Ischemia-GH: 76 (66, 85); Ischemia: 73 (67, 86); Control: 69 (58, 84)]. (**c**) Triglycerides [Ischemia-GH: 87 (38, 121); Ischemia: 108 (74, 115); Control: 64 (28, 88)]. (**d**) Low-density lipoprotein cholesterol [Ischemia-GH: 16 (12, 20); Ischemia: 17 (15, 20); Control: 16 (11, 24)].

No obvious changes were observed in lipid metabolism following GH administration. More specifically, at 8 weeks of age, there were no significant differences among the groups in total cholesterol (Figure 4a), high-density lipoprotein (Figure 4b), or low-density lipoprotein levels (Figure 4c). The triglyceride content in the Control group was significantly lower than in the Ischemia group [64 mg/dL (28, 88) vs. 108 mg/dL (74, 115), *p* = 0.034] (Figure 4d).

### 2.3. Metabolome Analyses 

**Figure 5:**
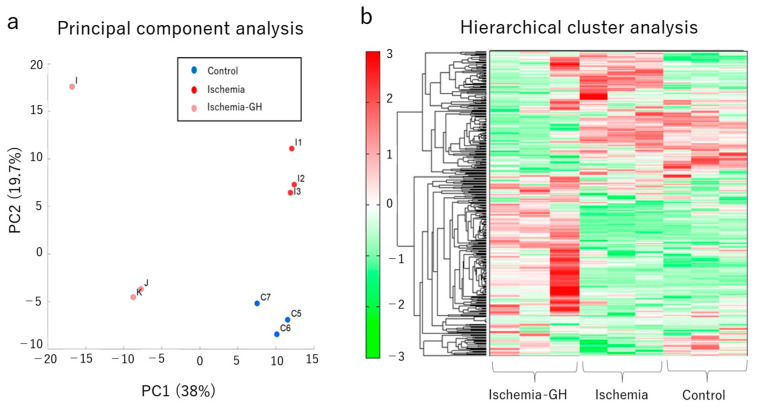
Metabolome analysis of liver (*n* = 3/group). For details on the principal component score, please refer to Tables S1 and S2. (**a**) Principal component analysis. (**b**) Heat map of the hierarchical cluster analysis.

**Figure 6:**
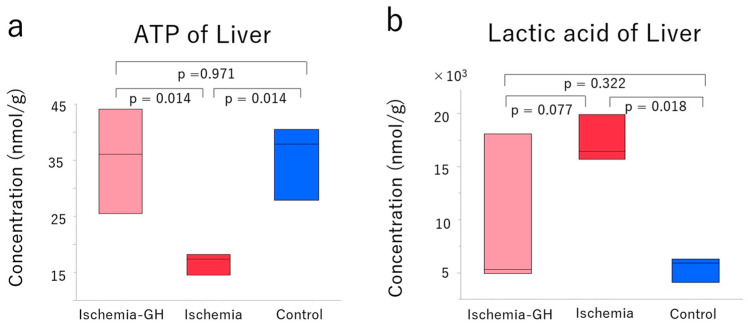
Adenosine triphosphate (ATP) and lactic acid level in the liver (*n* = 3/group) [median (min, max), nmol/g]. For the concentration of all metabolites of the liver, please refer to Table S3. (**a**) ATP [Ischemia-GH: 36.10 (25.51, 44.09); Ischemia: 17.39 nmol/g (14.53, 18.15); Control: 37.88 nmol/g (27.90, 40.53)]. (**b**) Lactic acid [Ischemia-GH: 5315 (4939, 18,093); Ischemia: 16,444 (15,724, 19,917); Control: 5911 (4095, 6285)].

The metabolome analysis of the liver showed that GH administration altered the metabolites. The principal component analysis (PCA) (Figure 5a) and hierarchical cluster analysis (HCA) (Figure 5b) of the metabolites revealed three trends, suggesting different metabolites. One metabolic pathway of interest was ATP production, which was significantly lower in the Ischemia group than in the other two groups [Ischemia: 17.39 nmol/g (14.53, 18.15) vs. Ischemia-GH: 36.10 nmol/g (25.51, 44.09), *p* = 0.014] [Ischemia: 17.39 nmol/g (14.53, 18.15) vs. Control: 37.88 nmol/g (27.90, 40.53), *p* = 0.014] (Figure 6a). The lactate level of the Control group was significantly lower than that of the Ischemia group [5911 nmol/g (4095, 6285) vs. 16,444 nmol/g (15,723, 19,916), *p* = 0.018]; the Ischemia-GH group showed a decreasing trend compared to the Ischemia group [5315 nmol/g (4939, 18,093) vs. 16,444 nmol/g (15,724, 19,917), *p* = 0.077] (Figure 6b).

Liver metabolome analysis indicated that GH administration reduced the expression of oxidative stress-related factors (Table 1). Several of these factors were significantly higher in the Ischemia group than in the Control group: 3-indoxylsulfuric acid [ratio 1.6, *p* = 0.024], cysteine [ratio 2.3, *p* = 0.032], and S-adenosylmethionine [ratio 1.6, *p* = 0.005]. Meanwhile, the abundance of S-adenosylmethionine was markedly lower in the Ischemia-GH group than in the Ischemia group [ratio 0.3, *p* = 0.004]. N,N-dimethylglycine was significantly decreased in the Ischemia-GH group compared with the Control group [ratio 0.5, *p* = 0.001]. Additionally, the abundance of the antioxidant hypotaurine was significantly increased in the Ischemia-GH group compared with the Control group [ratio 1.8, *p* = 0.041].

**Figure 7:**
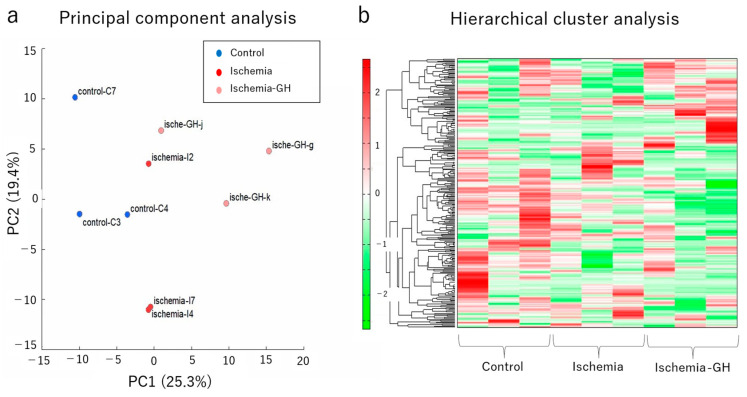
Metabolome analysis of muscle (*n* = 3/group). For details on the principal component score, please refer to Tables S4 and S5. (**a**) Principal component analysis. (**b**) Heat map of the hierarchical cluster analysis.

**Figure 8:**
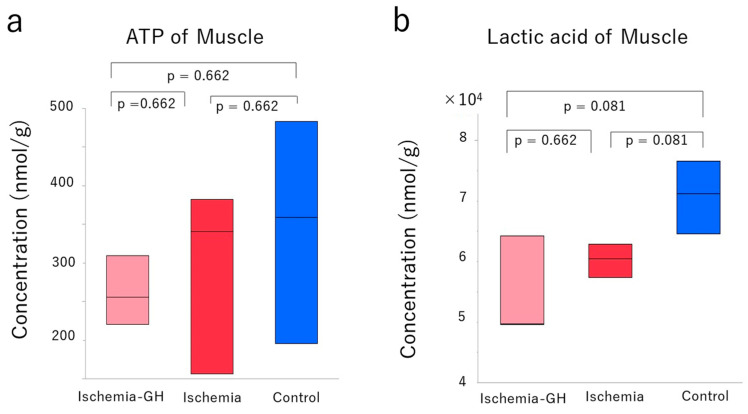
Adenosine triphosphate (ATP) and lactic acid level in the muscle (*n* = 3/group) [median (min, max), nmol/g]. For the concentration of all metabolites of the muscle, please refer to Table S6. (**a**) ATP [Ischemia-GH: 256 (220, 309); Ischemia: 341 (156, 382); Control: 359 (196, 483)]. (**b**) Lactic acid [Ischemia-GH: 49,693 (49,651, 64,223), Ischemia: 60,498 (57,337, 62,896); Control: 71,209 (64,641, 76,583)].

The metabolomic analysis of the muscle showed no consistent trend in metabolite changes (Figure 7a,b) and no significant differences in ATP production or lactate levels (Figure 8a,b). Moreover, there were no trends in oxidative stress-related factors following GH administration (Table 2).

### 2.4. Muscle Tissue Analysis

**Figure 9:**
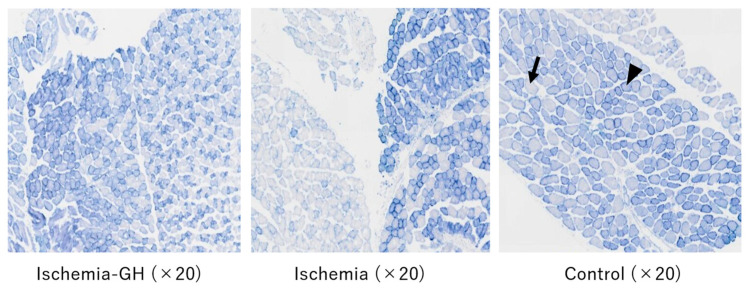
A fiber-type grouping of NADH-TR staining. All microscope images were taken at 20× magnification. The Ischemia group has a fiber-type grouping. Type 1 fibers are stained more intensely (arrowhead) than type 2 fibers (arrow).

Although no changes were observed in the muscle pathology based on hematoxylin and eosin (H&E) staining, changes in myofiber type were detected by nicotinamide adenine dinucleotide tetrazolium reductase (NADH-TR) staining (Figure 9). A mosaic-like distribution of type 1 and type 2 fibers was observed in the Control and Ischemia-GH groups. Meanwhile, the mosaic pattern disappeared in the Ischemia group; instead, the same muscle fibers were distributed in clusters, so-called fiber grouping (Figure 9). Compared to the other two groups, type 2 fibers comprised a significant proportion of the Ischemia group [Ischemia-GH vs. Ischemia, χ^2^(1) = 171.275, *p* < 0.001] [Control vs. Ischemia, χ^2^(1) = 165.651, *p* < 0.001] (Table 3). However, no significant differences in the ratio of type 1-to-type 2 fibers were detected between the Ischemia-GH and Control groups.

## 3. Discussion

A factor contributing to the pathogenesis of hyperglycemia in the non-obese hyperglycemic mouse model is the impaired mitochondrial function and increased myogenic insulin resistance caused by oxidative stressors [12]. In this study, we investigated whether GH administration could ameliorate the increase in myogenic insulin resistance.

### 3.1. Insulin Resistance Related to Skeletal Muscle

HOMA-IR is an indicator of insulin resistance, reflecting decreased insulin sensitivity in skeletal muscle, adipose tissue, and the liver [18]. Insulin resistance in skeletal muscle is said to be caused by impairments in the insulin signaling pathway. In this pathway, insulin binds to insulin receptors on the surface of skeletal muscle cell membranes, activating signaling molecules such as insulin receptor substrate (IRS), phosphoinositide 3-kinase (PI3K), and Akt. This activation leads to the translocation of vesicles containing glucose transporter 4 (GLUT4) to the cell membrane, facilitating glucose uptake [19,20]. Factors such as TNF-α and oxidative stress promote the serine phosphorylation of IRS-1, thereby inhibiting the interactions within the insulin signaling pathway [21,22,23,24].

GH treatment did not clearly improve body composition but did improve HOMA-IR. Moreover, H&E staining did not show structural changes in muscle tissue, whereas NADH-TR staining revealed altered myofiber types. Specifically, the Ischemia group had a significantly higher proportion of type 2 fibers than the other two groups. Skeletal muscle can be categorized into type 1 and type 2 fibers. Type 1 fibers have high aerobic metabolic capacity and are suitable for sustained exercise. In contrast, type 2 fibers have high anaerobic metabolic capacity and are suitable for rapid, high-force exertions. High insulin resistance is associated with a significantly lower proportion of type 1 fiber and a higher proportion of type 2 fiber [25,26]. In addition, the report on mice born stunted showed changes in muscle composition in adulthood associated with insulin resistance [27]. It is also speculated that the muscle composition in the Ischemia group affected insulin resistance.

The Ischemia-GH group had altered muscle composition due to GH administration, and the HOMA-IR improved significantly compared to the Ischemia group. Ayling et al. reported that type 1 fibers were reduced after pituitaryectomy but recovered to the baseline level after GH administration [28]. Loughna et al. also reported a decrease in myosin-heavy chains of type 1 fibers after pituitary removal, suggesting that GH is involved in the maintenance of myofiber type [29]. Although Aroniadou-Anderjaska et al. reported that GH administration had no effect on myofiber type changes [30], we found that type 1 fibers were significantly reduced in the Ischemia group compared to the Ischemia-GH group, indicating that GH administration did, in fact, influence myofiber type.

Fiber-type grouping was observed in the Ischemia group. Myofiber types are determined using the anterior horn cells of the spinal cord and are typically distributed in a mosaic-like pattern. However, when the innervating nerve is injured and the muscle is reinnervated, adjacent muscle fibers become innervated by the same spinal cord anterior horn cell. This results in fiber type grouping, in which muscle fiber types are clustered together [31,32]. Although previous reports have not examined the relationship between fiber-type grouping and insulin resistance, an association between fiber-type grouping and age-related sarcopenia has been suggested. Sarcopenia is caused by chronic inflammation and mitochondrial dysfunction, both affected by age-related oxidative stress [33]. Although muscle metabolome analysis did not reveal a significant decrease in oxidative stress markers, it has been reported that GH administration reduces oxidative stress [34,35]. Furthermore, GH acts on the liver to produce insulin-like growth factor-1 (IGF-1), an anabolic factor. In skeletal muscle, IGF-1 enhances protein synthesis, cell proliferation, and differentiation, with PI3K and Akt being well-known signaling pathways involved [19,20,36]. A decrease in IGF-1 has been suggested to accelerate sarcopenia progression [37]. Therefore, GH administration may have prevented fiber type grouping through IGF-1 mediation.

Although significant improvements in body composition were not observed with GH administration, the Ischemia-GH group showed a slight reduction in fat mass compared to the Ischemia group, albeit without statistical significance (Figure 4). TNF-α, secreted by adipocytes, is suggested to inhibit insulin signaling, thereby contributing to insulin resistance. In a real-life clinical setting, GH administration to children with small-for-gestational-age (SGA) short stature exacerbated insulin resistance during the first 3 months but improved insulin resistance after 2 years of treatment due to improved body composition [38]. Hence, the duration of GH administration affects body composition improvement. Indeed, another study reported that GH-induced improvements in body composition are related to duration and dosage [39]. In this study, we focused on young, non-obese T2DM and, therefore, dissected the animals for sample collection at 8 weeks of age. However, the body composition could have changed if GH was administered for a longer period. The trend towards a reduction in lean body mass with GH administration may have influenced insulin resistance.

In the present study, there were no adverse effects observed following GH administration related to serum lipoproteins. This agrees with previous study findings [15,16,40]. Moreover, we did not observe a worsening of lipid metabolism, suggesting that serum lipoproteins did not affect insulin resistance.

Metabolomic analysis of muscle did not reveal significant differences in ATP or lactic acid levels, indicating no clear improvement in mitochondrial function. The improvement in insulin resistance is likely attributed to the increase in type 1 muscle fibers and the avoidance of fiber-type grouping, rather than enhancements in mitochondrial function of muscle. 

### 3.2. Mitochondrial Dysfunction of Liver

Increased ATP production was observed in the Ischemia-GH group compared to the Ischemia group, suggesting an improved mitochondrial function of liver. The metabolites in the Ischemia-GH group differed from those in the Ischemia group in the PCA and HCA of the liver metabolome, particularly in terms of ATP and lactic acid. ATP production primarily occurs through glycolysis, the TCA cycle, and the respiratory chain. Among these, the respiratory chain produces the most ATP, generating a total of 34 moles, compared to 2 moles each from glycolysis and the TCA cycle. Mitochondria produce ATP through the TCA cycle and the respiratory chain in aerobic metabolism. When mitochondrial function is impaired, ATP production is reduced by changing anaerobic metabolism; lactic acid accumulates [41]. GH is protective by regulating the mitochondrial respiratory chain [42]. Although the lactic acid in the Ischemia-GH group did not differ significantly from the Ischemia group, there was a decreasing trend, suggesting an improved mitochondrial function of liver.

It is hypothesized that GH administration reduced oxidative stress-related factors and improved the mitochondrial function of liver in the Ischemia-GH group. Mitochondrial function is impaired by oxidative stressors [43,44]. In particular, oxidative stress-induced mitochondrial dysfunction occurs in a non-obese hyperglycemic mouse model after birth with LBW [12]. The Ischemia-GH group showed decreased oxidative stressors and increased antioxidants compared to the Ischemia group. Indeed, GH reportedly reduces oxidative stress [34,35]. 

### 3.3. Improvement of Insulin Resistance

**Figure 10:**
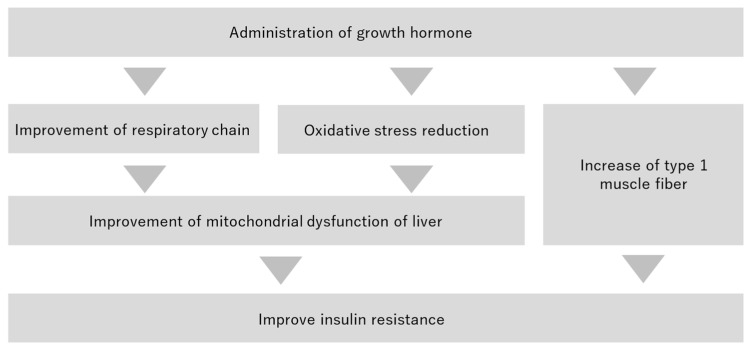
Potential theory for improved insulin resistance.

Two mechanisms are hypothesized for the improvement of insulin resistance (Figure 10). The first involves increasing type 1 muscle fiber, while the second focuses on the improved mitochondrial function of liver. In addition, two factors contribute to the improved mitochondrial function of liver: the protection of the mitochondrial respiratory chain, and a reduction in oxidative stress-related factors.

### 3.4. Limitations

This study had three limitations. First, the GH dose may have been too low. The therapeutic dose for human SGA short stature was used as a reference [45], but the improvement in insulin resistance due to GH is dose-dependent [39]. Second, we used a recombinant human GH. The report that oxidative stress is suppressed in mice treated with a recombinant human GH [35] was referenced, but there is no certification that a recombinant human GH is suitable for mice. Third, the administration period may have been too short. In clinical practice, when GH was administered to children with SGA short stature, insulin resistance worsened during the first 3 months but improved after 2 years of treatment due to improved body composition [41]. It has been suggested that improvement in body composition with GH is related to the treatment duration [42]; hence, long-term administration in this study might have made a clearer impact.

### 3.5. Future Directions

Future studies should assess whether increasing the dosage and prolonged treatment periods affect muscle metabolomics. Additionally, to clarify the role of GH in insulin resistance within muscle, it is essential to elucidate the underlying signaling pathways, the effects under increased GH dosages and prolonged treatment periods. 

GH formulations administered subcutaneously once a week have been approved for clinical use. Thus, it would be useful to validate the once-weekly formulation in terms of its potential for treatment.

## 4. Materials and Methods

### 4.1. Study Design and Protocol

**Figure 11:**
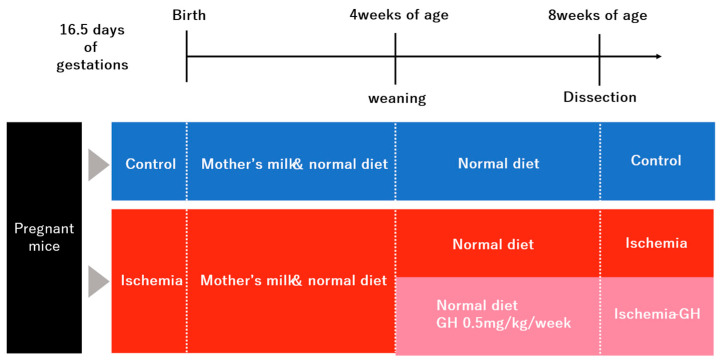
Experimental procedures. GH: growth hormone.

This study was conducted in accordance with the ARRIVE guidelines. The protocol was approved by the Animal Experiment Steering Committee of a Nihon University (Approval No.: AP21MED002-1, Approval date: 2 April 2021). The ICR strain of mice at day 12 of gestation was acquired from Sankyo Lab Service Co. (Tokyo, Japan). The mice were fed a standard solid diet (7.9% moisture, 5.1% lipid, 23.1% protein, 5.8% ash, 2.8% fiber, and 55.3% soluble constituents) from Oriental Yeast Co. (Tokyo, Japan), and provided water ad libitum. The fetuses were divided into two groups: one with Ischemia in utero (Ischemia group) and one without (Control group) (Figure 11).

**Figure 12:**
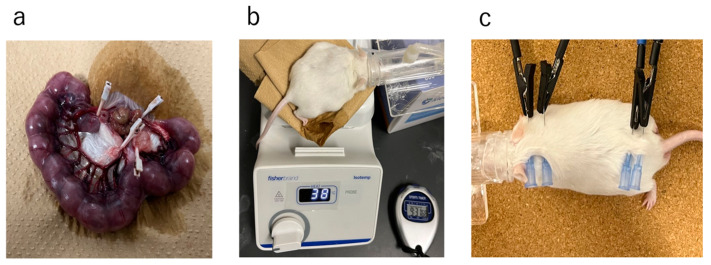
(**a**) Uterine artery ligation to induce intrauterine Ischemia. (**b**) Warming of a pregnant mouse at 37–38 °C on a hot plate. (**c**) Body composition measurement.

On gestational day 16.5, the lower abdomen was incised under isoflurane inhalation anesthesia (induction 3–4%, maintenance 2.5%). In the Ischemia group, the uterine artery was exposed, and the blood flow in the uterine artery was temporarily obstructed by clipping for 15 min (Figure 12a). During the interruption of blood flow, the fetuses were returned to the abdominal cavity, and kept warm on a hot plate at 37–38 °C (Figure 12b). After unclipping, the abdomen was sutured [12,46]. In the Control group, only a lower abdominal incision was made without exposing the uterine artery, and the abdomen was sutured after being kept warm on a hot plate at 37–38 °C for 15 min. Inhalation anesthesia was discontinued following the completion of all procedures.

Newborn pups were weaned at 4 weeks of age. At this time, females and males were separated; the female pups were selected in analyses. The Ischemia group was divided into a GH-treated group (Ischemia-GH group: *n* = 6) and a non-treated group (Ischemia group: *n* = 6). Growth hormone (Somatropin BS surepal, Sandoz Co., Ltd., Tokyo, Japan) was administered subcutaneously in the dorsal cervical region of the mice at a dose of 0.5 mg/kg/week, six times weekly until 8 weeks of age. The therapeutic dose for human SGA short stature was used as a reference [45,47]. All female pups were raised on a standard solid diet until 8 weeks of age.

Female pups were euthanized and dissected at 8 weeks of age. They were fasted for 12 h prior to dissection to avoid the effects of recent feedings on glucose metabolism. Body weight and composition were first measured (Figure 12c). The abdomen was incised under isoflurane inhalation anesthesia (induction 3–4%, maintenance 2.5%). Initially, blood was sampled from the heart, and they were euthanized, after which the liver and quadriceps muscles were harvested. The collected muscle tissue was freeze-fixed in liquid nitrogen at −80 °C. Similarly, the liver was frozen in liquid nitrogen and stored at −80 °C. Blood was centrifuged at room temperature and 3000 rpm for 5 min; the serum was collected and stored at −20 °C.

### 4.2. Body Weight and Body Composition Analyses

Body weights were measured at birth and at autopsy, and body composition was determined as fat mass and lean body mass (Figure 12c). The ImpediVET^TM^ (Bioresearch Center Co., Ltd., Nagoya, Japan) was used to measure body composition, and bioelectrical impedance analysis was employed to assess fat and lean body mass [48]. The body weight at autopsy and the percentage of each body component were recorded. Fat and lean body mass were calculated from the body weight at autopsy, and each weight percentage was as follows:

Fat mass (g) = body weight at autopsy (g) × percentage of fat mass/100

Fat-free mass (g) = body weight at autopsy (g) × percentage of fat-free mass/100

### 4.3. Glucose Metabolism and Serum Lipoprotein

To examine glucose and lipid metabolism, blood glucose, serum IRI, and serum lipid concentrations were measured at autopsy. Blood glucose levels were assessed using a Stat Strip WP2 (Nipro Co., Osaka, Japan) immediately after blood was sampled from the heart. Serum IRI and serum lipid concentrations were measured using a Mouse/Rat Total Insulin (high sensitivity) assay kit (Immuno-Biological Laboratories Co., Ltd., Fujioka, Gunma, Japan). Serum lipids were analyzed using gel filtration high-performance liquid chromatography (LipoSEARCH^®^; Skylight Biotech, Akita, Japan) [49,50,51]. 

The formula for calculating HOMA-IR was the human formula used in diabetes treatment, as there is no formula for mice [18]:

HOMA-IR = fasting blood glucose (mg/dL) × IRI (μIU/mL)/405

### 4.4. Metabolome Analysis in Liver and Muscle 

Metabolome analysis of the liver and muscle was performed to measure the metabolites. Frozen liver and muscle tissues were analyzed by Human Metabolome Technologies (HMT), Inc. (Yamagata, Japan).

Metabolome analysis was conducted using capillary electrophoresis time-of-flight mass spectrometry [52,53]. The spectrometer scanned from m/z 50 to 1000 and peaks were extracted using MasterHands (https://masterhands.in/ accessed on 1 September 2021), an automatic integration software (Keio University, Tsuruoka, Yamagata, Japan), obtaining peak information including m/z, peak area, and migration time (MT) [54]. Signal peaks corresponding to isotopomers, adduct ions, and other product ions of known metabolites were excluded, and the remaining peaks were annotated according to HMT’s metabolite database based on their m/z values and MTs. Areas of the annotated peaks were then normalized to internal standards and sample amounts to obtain relative levels of each metabolite. The primary 110 metabolites were quantified based on one-point calibrations using their respective standard compounds.

HCA and PCA were performed using HMT’s proprietary MATLAB and R programs, respectively [55,56]. 

### 4.5. Muscle Tissue Analyses 

Quadrate muscles were harvested and freeze-fixed at −80 °C with liquid nitrogen. H&E staining and NADH-TR staining were performed to examine changes in the muscle tissue. H&E staining was performed using paraffin embedding. NADH-TR staining required immersion in an NADH-TR reaction solution at 37 °C for 10 min and finally embedded in an optimal cutting temperature compound. Nitro TB and β-NADH (Fujifilm Wako Pure Chemical Co., Osaka, Japan) were used as the NADH-TR reaction solution.

A morphological comparison of the H&E staining of muscle tissue was performed. The distribution and ratio of type 1 fibers that stained dark and type 2 fibers that stained light in NADH-TR staining were examined.

### 4.6. Statistical Analyses 

Data for various metabolite analyses, including body weight, body composition, and metabolome analysis, were presented in box-and-whisker plots, and described using median (minimum, maximum) values. Statistical analysis was performed using JMP ver. 14 (SAS Institute, Cary, NC, USA).

The Kruskal–Wallis test and the Steel–Dwass multiple comparison test were applied to body weight, body composition, glucose, and lipid metabolites (*n* = 6/group), while the Student’s *t*-test or Welch’s *t*-test was conducted for metabolome analysis (*n* = 3/group). Significant differences were established at *p* < 0.05.

A morphological comparison of the H&E staining of muscle tissue was performed: we prepared three slides per group and randomly captured images at 20× magnification. In each photograph, 1000 muscle fibers stained with NADH-TR were randomly counted in the transverse section and classified them into type 1 and type 2 fibers, followed by a chi-square test. 

## 5. Conclusions

We showed that the administration of recombinant human GH to a non-obese hyperglycemic mouse model after birth with LBW improves insulin resistance. The mechanism involves increasing type 1 muscle fiber and improving mitochondrial function.

## Figures and Tables

**Table 1:** Oxidative stress markers of the liver.

Major Category	Compound Name	Comparative Analysis					
		Ischemia-GH vs. Ischemia		Ischemia-GH vs. Control		Ischemia vs. Control	
		Ratio ^1^	*p*-Value ^2^	Ratio ^1^	*p*-Value ^2^	Ratio ^1^	*p*-Value ^2^
Anti-oxidant	Ascorbic acid	1.0	0.802	1.1	0.566	1.1	0.184
Anti-oxidant	Carnosine	1.3	0.451	1.1	0.740	0.9	0.684
Anti-oxidant	Hypotaurine	1.4	0.076	1.8	0.041 *	1.3	0.359
Oxidative stress	3-Indoxylsulfuric acid	0.8	0.175	1.2	0.296	1.6	0.024 * (0.024 *)
Oxidative stress	Cysteine	3.1	0.265	6.8	0.188	2.3	0.032 * (0.032)
Oxidative stress	Methionine sulfoxide	1.2	0.723	1.4	0.564	1.2	0.444
Oxidative stress	*N*,N-Dimethylglycine	0.5	0.109	0.5	0.001 **	0.9	0.677
Oxidative stress	S-Adenosylmethionine	0.3	0.004 **	0.6	0.055	1.6	0.005 **

^1^ The ratio of the detected mean values between the two groups. ^2^ Welch’s *t*-test (* *p* < 0.05, ** *p* < 0.01).

**Table 2:** Oxidative stress markers of the muscle.

Major Category	Compound Name	Comparative Analysis					
		Ischemia-GH vs. Ischemia		Ischemia-GH vs. Control		Ischemia vs. Control	
		Ratio ^1^	*p*-Value ^2^	Ratio ^1^	*p*-Value ^2^	Ratio ^1^	*p*-Value ^2^
Anti-oxidant	Ascorbic acid	1.3	0.333	0.9	0.712	0.7	0.177
Anti-oxidant	Carnosine	0.9	0.762	0.8	0.517	0.9	0.822
Anti-oxidant	Hypotaurine	1.1	0.745	1.1	0.792	1.0	0.829
Oxidative stress	3-Indoxylsulfuric acid	0.8	N.A.	0.9	N.A.	1.1	0.593
Oxidative stress	Cysteine	1.6	0.638	1.1	0.948	0.7	0.753
Oxidative stress	Methionine sulfoxide	0.7	0.104	0.6	0.153	0.9	0.749
Oxidative stress	*N*,N-Dimethylglycine	0.9	0.306	1.1	0.426	1.2	0.004 *
Oxidative stress	S-Adenosylmethionine	0.9	0.593	0.9	0.396	1.0	0.718

^1^ The ratio of the detected mean values between the two groups. ^2^ Welch’s *t*-test (* *p* < 0.01). N.A.; not applicable

**Table 3:** Muscle fiber types.

	Type 1 Fibers	Type 2 Fibers	Total
Ischemia-GH	578	422	1000
Ischemia	288	712	1000
Control	573	427	1000
Total	1439	1561	3000

Chi-square test for each pair. Ischemia-GH vs. Ischemia (χ^2^(1) = 171.275, *p* < 0.001). Control vs. Ischemia (χ^2^(1) = 165.651, *p* < 0.001). Control vs. Ischemia-GH (χ^2^(1) = 0.051, *p* = 0.821).

## Data Availability

The data that support the findings of this study are available from the corresponding author upon reasonable request.

## Supplementary Materials

The following supporting information can be downloaded at: https://www.mdpi.com/article/10.3390/ijms25126294/s1.
