# Supplementary material for: Efficacy of Human Recombinant Growth Hormone in Females of a Non-Obese Hyperglycemic Mouse Model after Birth with Low Birth Weight
Source: Int J Mol Sci. 2024 Jun 7;25(12):6294. doi: 10.3390/ijms25126294 (PMC11203808; doi:10.3390/ijms25126294)
Supplement: Supplementary file 1 [file ijms-25-06294-s001.zip › Supplementary Table S1, PC of liver.pdf]

Supplementary Table S1. Principle component score of liver

contribution rate(%)		Ischemia-GH			Ischemia			Control		
		K	J	I	I1	I2	I3	C5	C6	C7
PC1	38.00	-8.74	-7.72	-16.79	12.11	12.43	11.94	11.57	10.15	7.55
PC2	19.66	4.55	3.70	-17.57	-11.07	-7.28	-6.44	6.93	8.45	5.20
PC3	9.94	-1.28	0.71	-10.78	7.66	5.43	0.77	-7.05	-5.92	-4.87
PC4	6.20	5.92	5.21	-1.80	1.27	1.16	-1.90	-4.00	-1.04	0.92
PC5	5.42	4.52	5.45	-1.02	-6.67	5.87	4.78	0.01	-3.72	-2.07
PC6	4.90	-6.36	1.94	-0.56	-5.53	0.05	6.54	0.00	2.27	-3.49
PC7	3.92	2.56	-1.48	0.35	1.83	-1.69	0.89	5.90	-0.35	-9.23
PC8	3.50	-5.47	6.72	1.35	1.76	-0.46	-1.81	-2.34	3.57	-1.69
PC9	3.10	-1.68	4.73	-0.63	1.60	1.07	-4.79	5.45	-2.21	-0.94
PC10	2.84	3.74	0.60	-0.78	1.71	-1.73	0.55	-3.06	6.29	-3.40
PC11	2.54	-0.30	-2.88	1.12	-2.61	6.85	-4.76	-0.55	3.25	-1.88
