# Supplementary material for: Efficacy of Human Recombinant Growth Hormone in Females of a Non-Obese Hyperglycemic Mouse Model after Birth with Low Birth Weight
Source: Int J Mol Sci. 2024 Jun 7;25(12):6294. doi: 10.3390/ijms25126294 (PMC11203808; doi:10.3390/ijms25126294)
Supplement: Supplementary file 1 [file ijms-25-06294-s001.zip › Supplementary Table S2, PC score of liver.pdf]

Supplementary Table S2. Metabolites and principle component score of liver

ID	HMT DB †			*m/z*	MT/RT	PC1	PC2
	Compound name	PubChem CID	HMDB ID				
A_0002	Propionic acid	1032	HMDB0000237	73.030	8.85	-8.9E-03	5.6E-02
A_0004	Pyruvic acid	1060	HMDB0000243	87.009	10.18	-2.6E-03	2.0E-02
A_0005	Butyric acid<br>Isobutyric acid	264<br>6590	HMDB0000039<br>HMDB0001873	87.045	8.09	3.9E-02	4.2E-03
A_0006	Lactic acid	612	HMDB0000190,<br>HMDB0001311	89.024	8.76	2.4E-02	-1.1E-01
A_0007	Isovaleric acid<br>DL-2-Methylbutyric Acid<br>Valeric acid	10430<br>8314<br>7991	HMDB0000718<br>HMDB0002176<br>HMDB0000892	101.061	7.66	3.0E-02	4.5E-02
A_0008	3-Hydroxybutyric acid	441	HMDB0000011,<br>HMDB0000357,<br>HMDB0000442	103.040	7.84	-3.9E-02	6.5E-02
A_0009	2-Hydroxybutyric acid	440864	HMDB0000008	103.040	8.03	-3.8E-02	7.7E-02
A_0010	Fumaric acid	444972	HMDB0000134	115.004	16.90	-3.0E-02	-9.9E-02
A_0012	Hexanoic acid	8892	HMDB0000535	115.076	7.38	6.7E-02	-2.6E-02
A_0013	*N*-Acetylglycine	10972	HMDB0000532	116.035	7.83	-9.6E-03	-4.6E-02
A_0014	Succinic acid	1110	HMDB0000254	117.019	14.93	-4.5E-02	-1.8E-02
A_0015	β-Hydroxyisovaleric acid	69362	HMDB0000754	117.055	7.55	-6.3E-02	-5.0E-03
A_0016	5-Hydroxypentanoic acid	25945	HMDB0061927	117.056	7.34	-3.3E-02	1.7E-02
A_0017	Isethionic acid	7866	HMDB0003903	124.991	9.28	1.6E-02	7.3E-02
A_0018	XA0003			124.991	9.95	-3.5E-02	-8.5E-03
A_0019	5-Oxoproline	7405	HMDB0000267	128.036	7.78	-7.7E-02	3.8E-02
A_0020	Heptanoic acid	8094	HMDB0000666	129.093	7.15	4.8E-02	-1.6E-02
A_0021	N-Acetylalanine-1<br>N-Acetyl-β-alanine-1	88064<br>76406	HMDB0000766	130.051	7.44	-4.4E-02	3.3E-02
A_0022	N-Acetylalanine-2<br>N-Acetyl-β-alanine-2	88064<br>76406	HMDB0000766	130.052	7.32	-1.1E-02	-9.6E-02
A_0023	6-Hydroxyhexanoic acid	14490		131.073	7.04	-8.0E-03	1.9E-02
A_0024	Malic acid	525	HMDB0000156,<br>HMDB0000744	133.014	15.14	-3.3E-02	-9.6E-02
A_0025	Threonic acid	5460407	HMDB0000943	135.030	7.61	5.8E-02	3.2E-02
A_0026	*p*-Toluic acid<br>*o*-Toluic acid<br>*m*-Toluic acid	7470<br>8373<br>7418		135.045	7.56	-8.0E-02	-7.1E-04
A_0027	Ethanolamine phosphate	1015	HMDB0000224	140.012	6.65	-4.1E-02	8.5E-02
A_0028	Octanoic acid	379	HMDB0000482	143.108	6.95	6.6E-02	2.0E-02
A_0029	4-Acetamidobutanoic acid	18189	HMDB0003681	144.067	7.12	-3.6E-04	7.8E-02
A_0031	2-Hydroxyglutaric acid	43	HMDB0000606,<br>HMDB0000694	147.030	12.84	4.5E-02	-3.0E-02
A_0032	3-Phenylpropionic acid	107	HMDB0000764	149.061	7.28	-6.4E-02	3.2E-02
A_0033	Oxypurinol	4644	HMDB0000786	151.027	6.01	-3.3E-02	3.2E-03
A_0034	Cysteinesulfinic acid	1549098	HMDB0000996	152.003	7.80	-6.8E-02	3.3E-02
A_0035	Pelargonic acid	8158	HMDB0000847	157.123	6.78	7.3E-02	3.1E-02
A_0036	2-Oxoadipic acid	71	HMDB0000225	159.031	13.17	-3.8E-02	2.4E-02
A_0037	Pimelic acid	385	HMDB0000857	159.067	10.85	-3.3E-02	1.7E-02
A_0038	8-Hydroxyoctanoic acid-2<br>2-Hydroxyoctanoic acid-2	69820<br>94180	HMDB0000711	159.102	6.77	-3.3E-02	1.7E-02
A_0039	8-Hydroxyoctanoic acid-1<br>2-Hydroxyoctanoic acid-1	69820<br>94180	HMDB0000711	159.103	6.70	-3.1E-02	1.2E-02
A_0040	3-Hydroxy-3-methylglutaric acid	1662		161.045	12.00	-6.3E-03	-5.1E-02
A_0041	Terephthalic acid	7489	HMDB0002428	165.019	12.58	2.1E-02	4.6E-03
A_0042	Perillic acid	1256	HMDB0004586	165.092	6.77	-6.0E-02	-5.9E-03

A_0043	XA0012			166.018	7.71	-4.3E-02	5.8E-02
A_0044	Phosphoenolpyruvic acid	1005	HMDB0000263	166.975	15.02	-7.3E-02	4.8E-02
A_0045	Uric acid	1175	HMDB0000289	167.021	7.33	-4.1E-02	8.4E-02
A_0046	Dihydroxyacetone phosphate	668	HMDB0001473	168.991	10.12	6.4E-02	-6.7E-02
A_0047	Glyceraldehyde 3-phosphate	729	HMDB0001112	168.992	9.39	5.0E-02	-4.5E-02
A_0048	Glycerol 3-phosphate	439162	HMDB0000126	171.006	9.71	3.2E-02	7.1E-04
A_0049	Decanoic acid	2969	HMDB0000511	171.140	6.64	-2.9E-02	6.6E-02
A_0050	Isovalerylalanine-2<br>N-Acetylleucine-2	129285<br>70912	HMDB0000747<br>HMDB0011756	172.098	6.74	-2.8E-02	-2.4E-02
A_0051	Isovalerylalanine-1<br>N-Acetylleucine-1	129285<br>70912	HMDB0000747<br>HMDB0011756	172.098	6.67	-4.9E-02	-5.1E-02
A_0052	*cis* -Aconitic acid	643757	HMDB0000072	173.011	17.74	4.1E-02	-4.4E-02
A_0053	*N* -Acetylasparagine	99715	HMDB0006028	173.056	6.96	5.8E-02	-7.2E-02
A_0054	Formiminoglutamic acid	439233	HMDB0000854	173.057	6.76	-7.7E-02	2.0E-02
A_0055	Suberic acid	10457	HMDB0000893	173.082	10.26	-3.4E-02	1.2E-02
A_0056	*N* -Acetylaspartic acid	65065	HMDB0000812	174.041	11.38	4.5E-02	2.2E-02
A_0057	Ascorbic acid	54670067	HMDB0000044	175.024	7.02	-1.5E-02	4.6E-02
A_0058	Allantoic acid	203	HMDB0001209	175.047	7.17	-4.2E-02	6.2E-03
A_0059	Hippuric acid	464	HMDB0000714	178.052	6.99	-6.1E-02	-3.8E-02
A_0060	Homovanillic acid<br>Hydroxyphenyllactic acid	1738<br>9378	HMDB0000118<br>HMDB0000755	181.051	6.86	2.9E-02	-2.5E-02
A_0061	4-Pyridoxic acid	6723	HMDB0000017	182.045	7.24	-7.7E-02	-3.9E-02
A_0062	3-Phosphoglyceric acid	439183	HMDB0000807	184.985	14.28	-8.0E-02	1.4E-02
A_0063	2-Phosphoglyceric acid	439278	HMDB0003391	184.986	14.01	-7.2E-02	-7.3E-04
A_0064	XA0017			186.114	6.54	-5.5E-02	5.4E-02
A_0065	*N* -Acetylglutamine	25561	HMDB0006029	187.073	6.72	3.3E-02	-9.5E-02
A_0066	Azelaic acid	2266	HMDB0000784	187.098	9.79	-3.4E-02	1.7E-02
A_0067	10-Hydroxydecanoic acid	74300		187.134	6.39	-3.3E-02	1.7E-02
A_0068	*N* -Acetylglutamic acid	70914	HMDB0001138	188.057	10.49	-4.0E-02	-4.7E-02
A_0069	*N* -Acetylmethionine	448580	HMDB0011745	190.056	6.75	3.0E-02	-8.7E-02
A_0071	Citric acid	311	HMDB0000094	191.020	17.19	3.2E-02	-7.6E-02
A_0072	XA0019			191.020	6.90	3.4E-02	1.3E-02
A_0073	Quinic acid	6508	HMDB0003072	191.054	6.72	-4.3E-02	5.9E-03
A_0074	Phenaceturic acid	68144	HMDB0000821	192.066	6.81	1.9E-02	-6.0E-02
A_0075	*N* -(*o* -Toluoyl)glycine	91637	HMDB0011723	192.067	6.76	-5.4E-02	6.5E-02
A_0076	Galacturonic acid-2<br>Glucuronic acid-2	439215<br>94715	HMDB0002545<br>HMDB0000127	193.036	6.73	2.6E-02	-3.5E-02
A_0077	Galacturonic acid-1<br>Glucuronic acid-1	439215<br>94715	HMDB0002545<br>HMDB0000127	193.036	6.87	1.8E-02	-8.4E-02
A_0078	Gluconic acid	10690	HMDB0000625	195.051	6.81	-9.4E-03	7.7E-02
A_0080	Lauric acid	3893	HMDB0000638	199.171	6.40	3.0E-02	7.8E-02
A_0081	Sebacic acid	5192	HMDB0000792	201.113	9.39	-3.7E-02	1.1E-02
A_0082	Indole-3-lactic acid<br>5-Methoxyindoleacetic acid	676157<br>18986	HMDB0000671<br>HMDB0004096	204.067	6.67	-6.6E-02	-4.2E-02
A_0083	*N* -Acetylphenylalanine	74839	HMDB0000512	206.084	6.62	-2.1E-02	-3.2E-02
A_0084	Glucaric acid	33037	HMDB0000663	209.029	11.38	-4.1E-02	5.7E-02
A_0085	Mucic acid	3037582	HMDB0000639	209.030	11.05	-6.7E-02	2.5E-02
A_0086	3-Indoxylsulfuric acid	10258	HMDB0000682	212.002	7.83	1.8E-02	-8.1E-02
A_0087	Pantothenic acid	6613	HMDB0000210	218.104	6.39	-7.8E-02	-1.8E-02
A_0088	Ethyl glucuronide	18392195	HMDB0010325	221.066	6.48	-2.9E-02	2.1E-02
A_0089	Myristoleic acid	5281119	HMDB0002000	225.186	6.25	-1.0E-02	8.8E-02
A_0090	Myristic acid	11005	HMDB0000806	227.202	6.22	2.8E-02	2.8E-02
A_0091	Ribose 5-phosphate	439167	HMDB0001548	229.011	8.58	7.1E-02	-5.6E-02

A_0092	Ribulose 5-phosphate	439184	HMDB0000618	229.012	8.90	8.2E-02	-1.6E-02
A_0093	XA0033			242.080	6.35	2.1E-02	-3.8E-02
A_0094	XA0080	440992		243.028	8.46	-6.6E-02	1.5E-03
A_0095	γ-Glu-Taurine	68759	HMDB0004195	253.051	6.72	-6.7E-02	-3.5E-02
A_0096	Ascorbate 2-sulfate	54676864		254.982	11.22	1.2E-02	-8.3E-03
A_0097	XA0035			254.982	10.74	3.8E-02	-3.8E-02
A_0098	Glucosamine 6-phosphate	440997	HMDB0001254	258.037	7.31	2.6E-03	-5.5E-02
A_0099	*myo*-Inositol 2-phosphate	160886		259.022	8.54	2.2E-02	-3.9E-02
A_0100	Glucose 1-phosphate	65533	HMDB0001586	259.023	8.27	3.6E-02	-7.4E-02
A_0101	Glucose 6-phosphate	5958	HMDB0001401	259.023	8.05	2.7E-02	-5.1E-02
A_0102	Fructose 6-phosphate	603	HMDB0000124	259.023	8.14	3.7E-02	-5.1E-02
A_0103	*myo*-Inositol 1-phosphate<br>*myo*-Inositol 3-phosphate	107737<br>440194	HMDB0000213<br>HMDB0006814	259.023	8.39	3.7E-02	6.3E-02
A_0104	2,3-Diphosphoglyceric acid	186004	HMDB0001294	264.954	13.71	2.0E-03	9.1E-02
A_0105	6-Phosphogluconic acid	91493	HMDB0001316	275.019	11.44	-6.7E-02	-5.3E-02
A_0106	Xanthosine	64959	HMDB0000299	283.070	6.35	-5.7E-02	-4.1E-02
A_0107	Orotidine	92751	HMDB0000788	287.054	6.56	6.8E-03	3.0E-02
A_0108	Sedoheptulose 7-phosphate	165007	HMDB0001068	289.033	7.88	-6.3E-02	4.6E-02
A_0109	*N*-Acetylglucosamine 6-phosphate	440996	HMDB0001062	300.048	7.54	-5.7E-02	-3.3E-02
A_0110	*N*-Acetylglucosamine 1-phosphate	440272	HMDB0001367	300.048	7.84	-7.7E-02	2.0E-02
A_0111	cCMP<br>2',3'-cCMP	19236<br>68934	 HMDB0011691	304.035	6.34	-3.7E-02	1.4E-02
A_0112	dCMP	13945	HMDB0001202	306.051	7.96	-4.3E-02	6.3E-02
A_0113	*N*-Acetylneuraminic acid	439197	HMDB0000230	308.099	6.02	-3.0E-02	-1.3E-02
A_0114	Ribulose 1,5-diphosphate	123658		308.979	12.04	4.7E-02	8.8E-03
A_0116	3'-CMP	66535		322.042	8.01	-2.8E-02	3.3E-03
A_0117	2'-CMP	101544	HMDB0011692	322.043	7.94	-2.8E-02	-6.4E-02
A_0118	CMP	6131	HMDB0000095	322.043	7.85	3.8E-02	-6.3E-02
A_0119	UMP	6030	HMDB0000288	323.029	7.98	7.5E-02	-2.0E-02
A_0120	*N*-Glycolylneuraminic acid	440001	HMDB0000833	324.094	6.00	-1.2E-02	-3.2E-02
A_0122	Ascorbate 2-glucoside	54693473		337.077	5.98	-3.4E-03	-1.1E-01
A_0123	Fructose 1,6-diphosphate	172313	HMDB0001058	338.990	11.37	5.6E-02	-6.8E-02
A_0124	cGMP	24316	HMDB0001314	344.028	6.33	7.5E-03	-8.2E-02
A_0125	AMP	6083	HMDB0000045	346.056	7.60	7.8E-02	-2.0E-02
A_0126	3'-AMP	41211	HMDB0003540	346.057	7.97	-4.6E-02	-8.4E-02
A_0127	IMP	8582	HMDB0000175	347.041	7.78	7.8E-02	-1.2E-02
A_0128	Prostaglandin $F_{2α}$	5280363	HMDB0001139	353.233	5.77	-7.4E-02	-1.1E-02
A_0129	GMP	6804	HMDB0001397	362.052	7.50	7.8E-02	-1.8E-02
A_0130	XA0055			369.000	11.19	5.1E-02	-6.3E-02
A_0131	NADPH_divalent	5884		371.539	9.08	6.3E-02	-5.0E-02
A_0132	CoA_divalent	87642		382.548	8.60	8.2E-02	1.3E-02
A_0134	FAD_divalent	643975		391.570	6.54	7.6E-02	-1.6E-02
A_0136	CDP	6132	HMDB0001546	402.007	9.19	-5.0E-02	2.4E-02
A_0137	UDP	6031	HMDB0000295	402.992	9.31	6.1E-02	-2.8E-02
A_0138	Acetyl CoA_divalent	444493		403.552	8.34	7.3E-02	2.1E-02
A_0139	Cholic acid	221493	HMDB0000619	407.279	5.76	-5.2E-02	-1.1E-02
A_0140	Isobutyryl CoA_divalent	3036931		417.574	8.15	6.0E-02	7.3E-03
A_0141	Thiamine diphosphate	1132	HMDB0001372	423.031	6.59	5.1E-02	3.3E-02
A_0143	ADP	6022	HMDB0001341	426.025	8.78	7.2E-02	1.7E-02
A_0144	GDP	8977	HMDB0001201	442.016	8.59	7.8E-02	1.8E-02
A_0145	XA0065			445.054	5.81	2.2E-02	-8.2E-03
A_0146	FMN	643976	HMDB0001520	455.098	6.91	-4.3E-02	6.9E-02
A_0147	Adenylosuccinic acid	447145	HMDB0000536	462.069	10.81	6.5E-02	-5.9E-02

A_0148	Glycocholic acid	10140	HMDB0000138	464.301	5.64	-6.1E-03	-2.4E-02
A_0152	UTP	6133	HMDB0000285	482.960	9.96	-6.6E-02	2.9E-02
A_0153	CDP-choline	13804	HMDB0001413	487.101	5.69	3.6E-02	5.1E-02
A_0155	ATP	5957	HMDB0000538	505.990	9.40	-5.2E-02	8.5E-02
A_0156	GTP	6830	HMDB0001273	521.979	9.18	-6.0E-02	6.5E-02
A_0157	ADP-ribose	445794	HMDB0001178	558.067	7.02	7.6E-02	-3.9E-02
A_0158	UDP-galactose<br>UDP-glucose	23724458<br>8629	HMDB0000302<br>HMDB0000286	565.044	7.12	5.0E-02	-7.0E-02
A_0159	UDP-glucuronic acid	17473	HMDB0000935	579.027	8.91	5.0E-02	4.5E-02
A_0160	GDP-fucose<br>ADP-glucose	10918995<br>16500	HMDB0001095<br>HMDB0006557	588.079	6.88	3.4E-02	-8.5E-02
A_0161	GDP-mannose<br>GDP-glucose	18396<br>46173703	HMDB0001163<br>HMDB0003351	604.066	6.86	7.6E-02	-4.2E-02
A_0162	UDP-*N*-acetylgalactosamine<br>UDP-*N*-acetylglucosamine	23724461<br>445675	HMDB0000304<br>HMDB0000290	606.069	6.98	7.6E-02	-4.7E-02
A_0163	CMP-*N*-acetylneuraminate	448209	HMDB0001176	613.138	6.78	5.4E-02	-2.3E-02
A_0164	NAD^+	5893	HMDB0000902	662.107	5.50	3.3E-02	8.6E-02
A_0165	NADH	439153	HMDB0001487	664.122	6.77	5.4E-02	4.3E-02
A_0166	$NADP^+$	5886	HMDB0000217	742.072	7.67	4.3E-02	7.3E-02
C_0001	Trimethylamine	1146	HMDB0000906	60.081	4.72	-4.3E-02	-1.9E-02
C_0002	Urea	1176	HMDB0000294	61.040	18.24	-6.1E-02	-5.3E-02
C_0003	Ethanolamine	700	HMDB0000149	62.060	5.09	-5.5E-02	7.1E-02
C_0004	XC0001			72.080	5.11	3.6E-02	-6.2E-02
C_0005	Aminoacetone	215	HMDB0002134	74.060	5.51	-7.3E-02	-3.8E-02
C_0006	Gly	750	HMDB0000123	76.039	6.73	-7.8E-02	-4.0E-02
C_0007	Trimethylamine *N*-oxide	1145	HMDB0000925	76.076	5.30	3.9E-02	-7.5E-02
C_0008	4-Methylpyrazole	3406		83.060	5.86	-4.8E-02	-7.1E-02
C_0009	Putrescine	1045	HMDB0001414	89.107	3.76	6.7E-02	1.9E-02
C_0010	β-Ala	239	HMDB0000056	90.055	5.89	-6.1E-02	7.5E-02
C_0011	Sarcosine	1088	HMDB0000271	90.055	7.70	-2.7E-02	-3.1E-02
C_0012	Dimethylaminoethanol	7902	HMDB0032231	90.091	5.55	-6.2E-02	-2.3E-02
C_0013	Ala	602	HMDB0000161,<br>HMDB0001310	91.058	7.29	-6.2E-02	-7.9E-02
C_0014	Glycerol	753	HMDB0000131	93.055	19.10	-6.7E-02	4.8E-02
C_0015	Phenol	996	HMDB0000228	95.046	4.36	-3.0E-02	6.4E-02
C_0016	Homoserinelactone	73509		102.055	5.71	7.3E-02	-4.8E-02
C_0017	*N*,*N*-Dimethylglycine	673	HMDB0000092	104.071	8.93	6.9E-02	1.3E-03
C_0018	GABA	119	HMDB0000112	104.071	6.19	-5.7E-02	-7.0E-02
C_0019	2-Aminoisobutyric acid<br>2-Aminobutyric acid	6119<br>6657	HMDB0001906<br>HMDB0000452	104.071	7.80	-7.8E-02	-3.4E-02
C_0020	3-Aminoisobutyric acid	64956	HMDB0003911	104.071	6.31	-6.7E-02	-2.6E-02
C_0021	3-Aminobutyric acid	10932		104.071	6.39	-5.6E-02	1.3E-02
C_0022	Choline	305	HMDB0000097	104.107	5.49	-6.6E-02	4.3E-02
C_0023	2,3-Diaminopropionic acid	364	HMDB0002006	105.066	5.77	-7.2E-02	-4.3E-02
C_0024	Ser	617	HMDB0000187,<br>HMDB0003406	106.050	8.15	-6.1E-02	-7.0E-02
C_0025	Diethanolamine	8113	HMDB0004437	106.086	6.14	-6.3E-02	1.8E-02
C_0026	Hypotaurine	107812	HMDB0000965	110.027	15.06	-6.9E-02	-3.5E-02
C_0027	Cytosine	597	HMDB0000630	112.051	5.83	-3.7E-02	-6.7E-02
C_0028	Histamine	774	HMDB0000870	112.087	3.81	9.8E-03	3.5E-02
C_0029	Uracil	1174	HMDB0000300	113.034	19.12	-8.4E-02	4.6E-03
C_0030	Creatinine	588	HMDB0000562	114.066	5.85	-4.7E-02	-2.1E-02

C_0031	3-Amino-2-piperidone	5200225	HMDB0000323	115.086	6.11	-2.3E-02	-1.3E-02
C_0032	Pro	614	HMDB0000162, HMDB0003411	116.070	8.80	-8.0E-02	-3.1E-02
C_0033	Guanidoacetic acid	763	HMDB0000128	118.061	6.64	-1.3E-02	-5.7E-02
C_0034	Val	1182	HMDB0000883	118.086	8.10	-8.0E-02	-2.1E-02
C_0035	Betaine	247	HMDB0000043	118.086	9.20	-3.0E-02	4.5E-02
C_0036	5-Aminovaleric acid	138	HMDB0003355	118.086	6.43	4.1E-02	-7.0E-02
C_0037	2,4-Diaminobutyric acid	134490	HMDB0006284	119.081	5.55	-1.3E-02	-9.5E-02
C_0038	Homoserine	12647	HMDB0000719	120.065	8.20	-2.0E-02	-1.1E-01
C_0039	Thr	6288	HMDB0000167	120.065	8.58	-7.0E-02	-5.7E-02
C_0040	2-Methylserine	439656		120.066	8.44	1.7E-02	-8.8E-02
C_0041	Betaine aldehyde_+H_2O	249		120.102	5.97	-7.4E-02	3.9E-02
C_0042	Purine	1044	HMDB0001366	121.050	6.64	-8.1E-02	1.5E-02
C_0043	Anserine_divalent	112072		121.069	5.46	-3.6E-02	-4.3E-02
C_0044	Cys	594	HMDB0000574, HMDB0003417	122.027	9.26	-6.4E-02	-6.7E-02
C_0045	2-Amino-2-(hydroxymethyl)-1,3-propanediol	6503		122.082	6.65	2.7E-02	-4.8E-02
C_0046	Nicotinamide	936	HMDB0001406	123.055	5.96	-6.2E-02	-6.6E-02
C_0047	Picolinamide	15070		123.055	8.76	-3.8E-02	-7.7E-02
C_0048	Picolinic acid	1018	HMDB0002243	124.039	15.89	-5.5E-02	4.7E-02
C_0049	Nicotinic acid	938	HMDB0001488	124.039	8.16	-7.8E-02	-1.9E-02
C_0050	Taurine	1123	HMDB0000251	126.022	19.10	-3.3E-02	1.0E-01
C_0051	1-Methylhistamine	3614	HMDB0000898	126.103	3.93	2.1E-03	-1.1E-02
C_0052	3-Hydroxy-2-methyl-4-pyrone	8369	HMDB0030776	127.038	19.19	3.2E-03	-1.2E-01
C_0053	Imidazole-4-acetic acid	96215	HMDB0002024	127.050	6.46	-1.2E-02	-8.0E-02
C_0055	XC0016			129.066	7.06	4.7E-03	-9.8E-02
C_0056	4-Oxopyrrolidine-2-carboxylic acid	107541		130.050	8.94	-8.3E-02	-2.0E-03
C_0057	Pipecolic acid	439227	HMDB0000070, HMDB0000716, HMDB0005960	130.087	8.33	-3.0E-03	-7.9E-02
C_0058	*trans*-Glutaconic acid	5280498	HMDB0000620	131.034	19.78	2.6E-02	3.0E-02
C_0059	Hydroxyproline	5810	HMDB0000725	132.065	9.86	-2.7E-03	-4.8E-02
C_0060	*cis*-4-Hydroxyproline	440014	HMDB0006055	132.066	8.99	-7.8E-02	-1.1E-02
C_0061	3-Guanidinopropionic acid	67701		132.077	6.45	-2.5E-02	-4.4E-02
C_0062	Creatine	586	HMDB0000064	132.077	7.14	-6.5E-02	-1.6E-02
C_0063	Ile	791	HMDB0000172	132.102	8.26	-7.6E-02	-3.0E-02
C_0064	Asn	236	HMDB0000168, HMDB0033780	133.061	8.58	4.6E-02	-8.7E-02
C_0065	Gly-Gly	11163	HMDB0011733	133.061	6.73	-4.9E-02	-6.3E-02
C_0066	Ornithine	389	HMDB0000214, HMDB0003374	133.097	5.46	-7.7E-02	-1.9E-02
C_0067	Leu	857	HMDB0000687	133.105	8.36	-7.3E-02	-4.3E-02
C_0068	Thiaproline	9934		134.027	11.57	1.9E-02	-6.7E-02
C_0069	Asp	424	HMDB0000191, HMDB0006483	134.045	9.51	-7.8E-02	-1.9E-02
C_0070	Homocysteine	778	HMDB0000742	136.043	8.54	-8.1E-02	9.1E-04
C_0071	Adenine	190	HMDB0000034	136.062	6.13	-2.7E-02	2.8E-02
C_0072	Hypoxanthine	790	HMDB0000157	137.046	9.32	-8.4E-02	1.2E-02
C_0073	1-Methylnicotinamide	457	HMDB0000699	137.071	5.91	-3.9E-02	-1.7E-02
C_0074	Trigonelline	5570	HMDB0000875	138.055	8.53	-1.0E-02	-7.3E-02
C_0075	Anthranilic acid	227	HMDB0001123	138.055	8.91	-7.5E-02	2.4E-02
C_0076	Tyramine	5610	HMDB0000306	138.091	6.66	-6.7E-02	4.5E-02

C_0077	γ-Glu-Lys_divalent	65254		138.582	6.91	-4.7E-02	4.8E-04
C_0078	Urocanic acid	736715	HMDB0000301	139.050	6.67	-3.5E-02	4.6E-03
C_0079	1-Methyl-4-imidazoleacetic acid	75810	HMDB0002820	141.066	6.63	2.0E-03	-6.6E-02
C_0080	1*H*-Imidazole-4-propionic acid	10105257		141.066	6.53	-1.2E-02	-8.6E-02
C_0081	Ectoine	126041		143.081	7.60	-3.9E-04	-5.3E-02
C_0082	XC0029<br>Stachydrine	0<br>115244	HMDB0004827	144.101	9.36	4.9E-03	-3.4E-02
C_0083	4-Guanidinobutyric acid	500	HMDB0003464	146.092	6.67	2.9E-02	-8.9E-02
C_0084	γ-Butyrobetaine	134	HMDB0001161	146.117	6.51	1.6E-02	-7.7E-02
C_0085	Spermidine	1102	HMDB0001257	146.165	3.61	7.8E-02	-8.2E-03
C_0086	Gln	738	HMDB0000641,<br>HMDB0003423	147.076	8.78	7.5E-02	-1.1E-02
C_0087	Lys	866	HMDB0000182,<br>HMDB0003405	147.113	5.51	-7.3E-02	-4.9E-02
C_0088	2-Methylthiazolidine-4-carboxylic acid	160736		148.042	11.78	-2.4E-02	-8.7E-02
C_0089	*threo*-β-Methylaspartic acid	440064		148.059	10.14	-5.2E-02	4.4E-03
C_0090	Isoglutamic acid	73064		148.060	7.41	-7.2E-02	-1.9E-02
C_0091	*N*-Acetylserine	65249	HMDB0002931	148.060	20.11	-5.3E-02	-7.9E-02
C_0092	Glu	611	HMDB0000148,<br>HMDB0003339	149.063	8.94	-8.4E-02	5.3E-04
C_0093	Met	876	HMDB0000696	150.058	8.74	-7.8E-02	-2.7E-02
C_0094	Triethanolamine	7618		150.112	6.63	-2.7E-02	-8.5E-02
C_0095	Guanine	764	HMDB0000132	152.057	6.72	4.9E-02	4.8E-02
C_0096	Xanthine	1188	HMDB0000292	153.040	16.78	-8.0E-02	2.3E-02
C_0097	N^1-Methyl-4-pyridone-5-carboxamide	440810	HMDB0004194	153.066	15.69	-4.3E-02	2.7E-02
C_0098	4-(β-Acetylaminoethyl)imidazole	69602		154.098	6.78	7.6E-03	-4.9E-02
C_0099	His	773	HMDB0000177	156.077	5.87	-7.4E-02	-5.2E-02
C_0100	Imidazolelactic acid	793		157.060	7.12	-3.6E-02	-8.3E-02
C_0101	XC0145<br>Ala-Ala	15331<br>5460362	HMDB0003459	161.091	7.46	7.4E-02	-4.2E-02
C_0102	Tryptamine	1150	HMDB0000303	161.106	6.68	-5.6E-02	6.1E-02
C_0103	N^6-Methyllysine	164795	HMDB0002038	161.128	5.69	-5.9E-02	-6.9E-02
C_0104	*O*-Acetylhomoserine<br>2-Aminoadipic acid	439389<br>92136	HMDB0000510	162.076	8.94	-3.5E-02	4.5E-02
C_0105	Carnitine	85	HMDB0000062	162.112	6.85	-6.7E-02	-2.9E-02
C_0106	5-Hydroxylysine	3032849	HMDB0000450	163.107	5.73	-6.7E-02	9.1E-04
C_0107	Pterin	73000	HMDB0000802	164.057	8.51	-4.8E-02	-3.4E-02
C_0108	Methionine sulfoxide	158980	HMDB0002005	166.053	9.75	-5.0E-02	-5.0E-02
C_0109	7-Methylguanine	11361	HMDB0000897	166.071	6.64	-7.2E-02	-1.0E-02
C_0110	Phe	994	HMDB0000159	166.086	9.03	-6.5E-02	-5.5E-02
C_0111	Taurocyamine	68340	HMDB0003584	168.043	19.12	-6.6E-03	1.0E-01
C_0112	Pyridoxal	1050	HMDB0001545	168.065	7.07	-5.9E-02	-8.1E-03
C_0113	Tyr-Arg_divalent	123804		169.595	6.18	7.1E-02	-1.4E-02
C_0114	1-Methylhistidine<br>3-Methylhistidine	92105<br>64969	HMDB0000001<br>HMDB0000479	170.092	6.03	-7.3E-02	-9.8E-03
C_0115	XC0147	4173		172.072	7.82	4.5E-02	3.8E-02
C_0116	XC0040			174.088	9.85	4.8E-02	-4.2E-02
C_0117	*N*-Acetylornithine	439232	HMDB0003357	175.108	7.70	7.0E-02	-4.1E-02
C_0118	N^5-Ethylglutamine	439378		175.108	9.28	1.8E-02	-7.5E-02
C_0119	Arg	6322	HMDB0000517,<br>HMDB0003416	175.119	5.71	3.9E-02	-9.5E-02
C_0120	Guanidinosuccinic acid	439918	HMDB0003157	176.067	8.21	9.6E-03	-6.0E-02

C_0121	Diethylaminomalonic acid	81272		176.092	7.31	4.6E-03	-5.7E-02
C_0122	Citrulline	9750	HMDB0000904	176.103	9.02	-7.7E-02	4.0E-02
C_0123	Serotonin	5202	HMDB0000259	177.103	7.06	-2.2E-02	5.7E-02
C_0124	Gluconolactone	7027	HMDB0000150	179.055	19.91	-9.9E-03	7.8E-02
C_0125	Xanthopterin	8397		180.052	10.91	2.7E-02	7.7E-03
C_0126	Mannosamine	440049		180.087	7.24	-4.3E-02	-6.4E-02
C_0127	Glucosamine	439213	HMDB0001514	180.087	7.49	-5.2E-02	-8.9E-02
C_0128	Tyr	1153	HMDB0000158	182.081	9.26	-7.5E-02	-4.1E-02
C_0129	Phosphorylcholine	1014	HMDB0001565	184.074	17.46	1.9E-02	-3.8E-02
C_0130	N^1-Acetylspermidine	496	HMDB0001276	188.176	5.06	7.5E-02	-3.6E-03
C_0131	N^8-Acetylspermidine	123689	HMDB0002189	188.176	5.12	4.3E-02	2.1E-02
C_0132	Gly-Leu			189.123	7.79	4.4E-02	-2.2E-02
C_0133	*N*-Acetyllysine	92907	HMDB0000446	189.123	7.90	7.5E-02	-8.3E-03
C_0134	N^6-Acetyllysine	92832	HMDB0000206	189.123	9.33	-5.3E-02	-2.2E-02
C_0135	N_{ω}-Methylarginine	132862		189.134	5.98	-7.3E-02	-4.5E-02
C_0136	N^6,N^6,N^6-Trimethyllysine	440120	HMDB0001325	189.159	5.76	-3.2E-02	6.8E-02
C_0137	Homocitrulline	65072	HMDB0000679	190.119	9.11	8.3E-03	-7.7E-02
C_0138	Gly-Asp	97363		191.067	8.01	-7.5E-02	-4.1E-02
C_0139	2,6-Diaminopimelic acid	439283	HMDB0001370	191.103	7.18	-3.0E-02	-4.6E-02
C_0140	*N*-Acetylhistidine	75619		198.087	7.93	-1.1E-02	-9.7E-02
C_0141	Metanephrine	21100	HMDB0004063	198.111	7.60	-2.5E-02	5.9E-03
C_0142	ADMA	123831	HMDB0001539	203.150	6.15	-6.9E-02	-5.3E-02
C_0143	SDMA	169148	HMDB0003334	203.150	6.25	-8.1E-02	-2.3E-02
C_0144	Spermine	1103	HMDB0001256	203.223	3.58	5.9E-02	3.9E-02
C_0145	*O*-Acetylcarnitine	439756	HMDB0000201	204.123	7.28	3.6E-02	-3.8E-03
C_0146	γ-Glu-Gly	165527	HMDB0011667	205.082	9.88	-5.1E-02	-7.4E-02
C_0147	Trp	1148	HMDB0000929	205.097	8.97	-6.9E-02	-5.1E-02
C_0148	Carboxymethyllysine	123800		205.118	7.47	-1.9E-02	2.1E-03
C_0149	Lipoamide	863	HMDB0000962	206.066	19.49	-4.0E-02	7.1E-02
C_0150	Kynurenine	846	HMDB0000684	209.092	8.16	-8.3E-02	-8.5E-03
C_0151	Propionylcarnitine<br>XC0061	188824<br>0	HMDB0000824	218.138	7.52	-4.3E-02	-2.6E-02
C_0152	β-Ala-Lys	440638		218.149	5.42	6.7E-02	-5.8E-02
C_0153	γ-Glu-Ala	440103	HMDB0006248	219.097	10.10	-6.3E-02	-7.4E-02
C_0154	XC0065			221.092	10.77	-2.6E-02	2.1E-02
C_0155	*N*-Acetylglucosylamine	439454	HMDB0001104	221.113	8.02	-8.0E-02	1.9E-02
C_0156	*N*-Acetylgalactosamine<br>*N*-Acetylglucosamine<br>*N*-Acetylmannosamine	35717<br>439174<br>439281	HMDB0000853<br>HMDB0000215<br>HMDB0001129	222.097	19.12	-8.2E-02	1.5E-02
C_0157	Cystathionine	834	HMDB0000099	223.075	8.06	-6.4E-02	-6.2E-02
C_0158	Carnosine	439224	HMDB0000033	227.114	5.41	-9.7E-03	-1.1E-02
C_0159	2'-Deoxycytidine	13711	HMDB0000014	228.099	7.62	-6.8E-02	2.7E-02
C_0160	Ergothioneine	3037043	HMDB0003045	230.096	14.51	-2.5E-02	8.6E-02
C_0161	Butyrylcarnitine	439829	HMDB0002013	232.154	7.73	6.7E-02	4.7E-03
C_0162	Thr-Asp	3280446		235.092	8.62	6.2E-02	-6.2E-02
C_0163	γ-Glu-Ser	22844748	HMDB0029158	235.093	10.34	-5.6E-02	-4.9E-02
C_0164	Ser-Glu			235.093	8.43	2.4E-02	-8.5E-02
C_0165	7,8-Dihydrobiopterin	119055	HMDB0000038	240.109	9.00	-6.9E-02	-5.3E-02
C_0166	Homocarnosine	10243361	HMDB0000745	241.129	5.46	-4.9E-03	-2.8E-02
C_0167	Thymidine	5789	HMDB0000273	243.094	19.19	3.9E-02	-5.6E-02
C_0168	Cytidine	6175	HMDB0000089	244.093	7.82	-8.2E-02	1.3E-02
C_0169	Uridine	6029	HMDB0000296	245.077	19.16	-7.7E-02	-4.6E-02
C_0170	Isovalerylcarnitine	6426851	HMDB0000688	246.170	7.83	4.3E-02	6.2E-02
C_0171	γ-Glu-Val	7015683	HMDB0011172	247.129	10.44	-4.0E-02	-6.3E-02
C_0172	Malonylcarnitine	22833583	HMDB0002095	248.112	8.26	2.1E-02	-5.1E-02

C_0173	Pyridoxamine 5'-phosphate	1053	HMDB0001555	249.063	8.51	6.0E-02	1.3E-02
C_0174	γ-Glu-Thr	53861142	HMDB0029159	249.108	10.45	-5.1E-02	-5.4E-02
C_0175	γ-Glu-Cys	123938	HMDB0001049	251.070	10.53	-4.7E-02	-3.9E-02
C_0176	XC0089			255.098	7.68	-5.8E-02	6.6E-02
C_0177	XC0154	3182		255.107	19.13	-4.1E-02	-8.9E-02
C_0178	Glycerophosphocholine	439285	HMDB0000086	258.108	18.66	1.8E-02	-1.1E-01
C_0179	γ-Glu-Ile<br>γ-Glu-Leu	22885096<br>151023	HMDB0011170<br>HMDB0011171	261.143	10.61	-6.3E-02	-3.5E-02
C_0180	γ-Glu-Asn	131801686	HMDB0029144	262.103	10.49	-2.9E-02	-4.6E-03
C_0181	γ-Glu-Ornitine	189156	HMDB0002248	262.140	6.86	-5.8E-02	9.1E-03
C_0182	γ-Glu-Asp	161197	HMDB0030419	263.088	10.69	-4.8E-02	-5.1E-02
C_0183	Thiamine	1130	HMDB0000235	265.111	5.25	-6.9E-02	-5.5E-02
C_0184	Adenosine	60961	HMDB0000050	268.103	8.00	6.5E-02	3.1E-02
C_0185	Inosine	6021	HMDB0000195	269.088	16.73	-1.8E-02	1.6E-02
C_0186	γ-Glu-Gln	150914	HMDB0011738	276.118	10.70	5.5E-02	-6.8E-02
C_0187	Glu-Glu	439500		277.103	8.87	8.2E-03	-9.3E-02
C_0188	γ-Glu-Glu	92865	HMDB0011737	277.103	10.79	-7.5E-02	-3.4E-02
C_0189	Saccharopine	160556	HMDB0000279	277.140	8.75	-2.7E-02	8.8E-02
C_0190	*N*-(1-Deoxy-1-fructosyl)valine	131752247	HMDB0037844	280.140	11.55	-7.3E-02	-2.9E-02
C_0191	1-Methyladenosine	27476	HMDB0003331	282.117	8.06	-8.1E-02	-9.5E-03
C_0192	Guanosine	6802	HMDB0000133	284.099	10.44	6.6E-02	1.1E-02
C_0193	γ-Glu-His	7017195	HMDB0029151	285.118	7.03	-4.4E-02	-5.3E-02
C_0194	His-Glu	7010583		285.118	6.06	-6.1E-03	-9.9E-02
C_0195	Ophthalmic acid	7018721	HMDB0005765	290.133	10.87	-4.3E-02	-7.9E-02
C_0196	Argininosuccinic acid	16950	HMDB0000052	291.130	7.64	3.2E-02	-1.0E-01
C_0197	γ-Glu-Phe	111299	HMDB0000594	295.130	10.71	-5.6E-02	-2.1E-02
C_0198	5'-Deoxy-5'-methylthioadenosine	439176	HMDB0001173	298.097	8.17	7.6E-02	-1.8E-02
C_0199	N^1-Methylguanosine	96373	HMDB0001563	298.115	9.89	-8.2E-02	1.0E-02
C_0200	Arg-Glu			304.161	6.01	3.1E-02	-9.3E-02
C_0201	Glutathione (GSSG)_divalent	65359		307.085	9.99	4.2E-02	-4.1E-02
C_0202	Glutathione (GSH)	124886	HMDB0000125	309.096	10.90	-9.9E-03	-7.8E-02
C_0203	XC0126			310.115	12.44	-7.9E-02	5.8E-03
C_0204	Tyr-Glu			311.122	9.06	6.0E-02	-6.7E-02
C_0205	*S*-Methylglutathione	115260		322.106	11.01	2.4E-02	-6.9E-02
C_0206	XC0132			325.160	6.99	-5.1E-02	-1.9E-02
C_0207	Ile-Pro-Pro			326.205	8.92	-1.7E-02	-9.5E-02
C_0208	NMN	14180	HMDB0000229	335.064	17.59	4.7E-02	8.2E-02
C_0209	Thiamine phosphate	1131	HMDB0002666	345.078	8.68	-2.4E-02	8.4E-02
C_0210	XC0137			350.100	11.13	6.7E-02	5.3E-02
C_0211	Riboflavin	493570	HMDB0000244	377.147	19.00	-8.0E-02	2.3E-02
C_0212	*S*-Lactoylglutathione	440018	HMDB0001066	380.114	11.39	2.7E-02	8.8E-02
C_0213	*S*-Adenosylhomocysteine	439155	HMDB0000939	385.129	7.06	-5.8E-02	1.7E-02
C_0214	*S*-Adenosylmethionine	34755	HMDB0001185	399.144	5.72	7.0E-02	-5.9E-02
C_0215	Cysteine glutathione disulfide	10455148	HMDB0000656	427.098	9.55	-6.1E-02	-6.5E-02
C_0216	Tetrahydrofolic acid	135444742	HMDB0001846	446.178	9.75	-1.2E-02	-4.3E-02
C_0217	5-Methyltetrahydrofolic acid	444412	HMDB0001396	460.193	9.70	-3.2E-02	-3.5E-03

MT: migration time, RT: retention time
