# Supplementary material for: Efficacy of Human Recombinant Growth Hormone in Females of a Non-Obese Hyperglycemic Mouse Model after Birth with Low Birth Weight
Source: Int J Mol Sci. 2024 Jun 7;25(12):6294. doi: 10.3390/ijms25126294 (PMC11203808; doi:10.3390/ijms25126294)
Supplement: Supplementary file 1 [file ijms-25-06294-s001.zip › Supplementary Table S3, all metabolites of liver.pdf]

Supplementary Table S3. Concentrations of all metabolites of liver

ID	Metabolite	PubChem CID	HMDB ID	Concentration (nmol/g)								
				Ischemia-GH			Ischemia			Control		
				K	J	I	I1	I2	I3	C5	C6	C7
A_0009	2-Hydroxybutyric acid	440864	HMDB0000008	71	66	53	N.D.	42	50	70	83	42
A_0030	2-Oxoglutaric acid	51	HMDB0000208	N.D.	N.D.	N.D.	N.D.	N.D.	N.D.	N.D.	N.D.	N.D.
A_0011	2-Oxoisovaleric acid	49	HMDB0000019	N.D.	N.D.	N.D.	N.D.	N.D.	N.D.	N.D.	N.D.	N.D.
A_0063	2-Phosphoglyceric acid	439278	HMDB0003391	N.D.	14	14	N.D.	N.D.	N.D.	N.D.	N.D.	N.D.
A_0008	3-Hydroxybutyric acid	441	HMDB0000011, HMDB0000357, HMDB0000442	1,333	769	823	266	420	408	736	1,195	508
A_0062	3-Phosphoglyceric acid	439183	HMDB0000807	71	87	76	17	25	29	22	32	33
A_0105	6-Phosphogluconic acid	91493	HMDB0001316	117	114	157	113	100	75	33	49	45
A_0138	Acetyl CoA_divalent	444493		N.D.	N.D.	N.D.	0.4	0.7	0.6	1.3	1.0	1.0
C_0071	Adenine	190	HMDB0000034	8.8	6.2	9.1	8.7	7.2	7.2	7.4	6.9	12
C_0184	Adenosine	60961	HMDB0000050	57	26	23	75	51	51	66	88	76
A_0143	ADP	6022	HMDB0001341	57	71	66	96	102	120	142	154	102
C_0013	Ala	602	HMDB0000161, HMDB0001310	2,835	2,694	4,524	2,562	2,335	2,254	1,427	1,202	1,653
A_0125	AMP	6083	HMDB0000045	92	190	171	839	766	983	917	869	350
C_0075	Anthranilic acid	227	HMDB0001123	1.7	1.2	1.5	0.2	N.D.	0.4	0.2	0.2	0.4
C_0119	Arg	6322	HMDB0000517, HMDB0003416	1.3	2.2	4.1	6.9	5.8	4.9	2.3	1.8	2.1
C_0064	Asn	236	HMDB0000168, HMDB0033780	120	139	246	234	226	244	206	177	149
C_0069	Asp	424	HMDB0000191, HMDB0006483	1,517	1,468	1,717	647	678	601	424	563	497
A_0155	ATP	5957	HMDB0000538	44	36	26	15	17	18	28	41	38
C_0035	Betaine	247	HMDB0000043	1,451	1,065	1,323	1,065	996	1,082	1,132	1,588	1,692
C_0041	Betaine aldehyde_+H_2O	249		133	219	157	49	56	61	71	103	93
A_0121	cAMP	6076	HMDB0000058	N.D.	N.D.	N.D.	N.D.	N.D.	N.D.	N.D.	N.D.	N.D.
C_0158	Carnosine	439224	HMDB0000033	0.6	1.5	1.1	0.5	1.0	1.0	1.0	0.5	1.3
A_0136	CDP	6132	HMDB0001546	2.0	2.9	2.7	N.D.	N.D.	N.D.	1.1	1.8	N.D.
A_0124	cGMP	24316	HMDB0001314	2.0	2.7	3.4	4.3	3.4	3.7	1.5	3.2	1.4
C_0022	Choline	305	HMDB0000097	774	1,203	810	396	422	486	521	828	539
A_0052	*cis*-Aconitic acid	643757	HMDB0000072	N.D.	N.D.	N.D.	N.D.	8.5	7.9	N.D.	N.D.	N.D.
A_0071	Citric acid	311	HMDB0000094	96	140	193	136	173	161	101	110	153
C_0122	Citrulline	9750	HMDB0000904	61	55	51	34	33	36	36	41	45
A_0118	CMP	6131	HMDB0000095	33	47	77	80	58	72	60	78	62
A_0132	CoA_divalent	87642		82	59	28	115	121	128	132	131	116
C_0062	Creatine	586	HMDB0000064	391	409	416	207	174	257	240	163	274
C_0030	Creatinine	588	HMDB0000562	8.9	5.5	8.5	4.3	4.0	4.8	5.4	4.3	6.0
A_0151	CTP	6176	HMDB0000082	N.D.	N.D.	N.D.	N.D.	N.D.	N.D.	N.D.	N.D.	N.D.
C_0044	Cys	594	HMDB0000574, HMDB0003417	7.5	13	33	6.5	5.9	5.1	4.1	2.1	1.6
C_0168	Cytidine	6175	HMDB0000089	25	30	30	6.8	4.2	4.4	6.6	9.6	13
C_0027	Cytosine	597	HMDB0000630	0.4	0.5	0.8	0.3	0.4	0.3	N.D.	N.D.	0.4
A_0154	dATP	15993	HMDB0001532	N.D.	N.D.	N.D.	N.D.	N.D.	N.D.	N.D.	N.D.	N.D.
A_0149	dCTP	65091	HMDB0000998	N.D.	N.D.	N.D.	N.D.	N.D.	N.D.	N.D.	N.D.	N.D.
A_0046	Dihydroxyacetone phosphate	668	HMDB0001473	12	34	108	660	485	448	146	192	171
A_0135	dTDP	164628	HMDB0001274	N.D.	N.D.	N.D.	N.D.	N.D.	N.D.	N.D.	N.D.	N.D.
A_0115	dTMP	9700	HMDB0001227	N.D.	N.D.	N.D.	N.D.	N.D.	N.D.	N.D.	N.D.	N.D.
A_0150	dTTP	64968	HMDB0001342	N.D.	N.D.	N.D.	N.D.	N.D.	N.D.	N.D.	N.D.	N.D.
A_0079	Erythrose 4-phosphate	122357	HMDB0001321	N.D.	N.D.	N.D.	N.D.	N.D.	N.D.	N.D.	N.D.	N.D.
A_0123	Fructose 1,6-diphosphate	172313	HMDB0001058	N.D.	N.D.	4.4	408	285	249	30	23	42
A_0102	Fructose 6-phosphate	603	HMDB0000124	48	57	85	390	94	63	66	102	114
A_0010	Fumaric acid	444972	HMDB0000134	472	589	1,028	805	742	816	227	321	315
C_0018	GABA	119	HMDB0000112	47	61	260	36	36	43	23	25	19
A_0144	GDP	8977	HMDB0001201	14	14	11	22	22	21	27	28	24
C_0086	Gln	738	HMDB0000641, HMDB0003423	933	1,073	408	2,051	2,505	2,018	1,658	1,616	1,231
C_0092	Glu	611	HMDB0000148, HMDB0003339	3,519	3,463	4,264	1,399	1,289	1,232	1,412	1,790	1,532
A_0078	Gluconic acid	10690	HMDB0000625	664	874	739	582	457	632	1,000	1,066	765
A_0100	Glucose 1-phosphate	65533	HMDB0001586	22	25	55	248	94	69	23	29	28
A_0101	Glucose 6-phosphate	5958	HMDB0001401	149	136	200	1,728	241	124	161	170	160
C_0202	Glutathione (GSH)	124886	HMDB0000125	3,058	3,005	3,137	4,309	3,745	3,200	1,964	2,026	1,933
C_0201	Glutathione (GSSG)_divalent	65359		385	490	638	871	730	652	615	708	797
C_0006	Gly	750	HMDB0000123	2,457	2,420	2,967	2,090	1,887	1,824	1,493	1,575	1,617
A_0047	Glyceraldehyde 3-phosphate	729	HMDB0001112	N.D.	N.D.	N.D.	24	25	N.D.	14	N.D.	N.D.

A_0048	Glycerol 3-phosphate	439162	HMDB0000126	1,728	2,396	2,031	2,029	2,274	2,196	2,015	2,490	2,037
A_0003	Glycolic acid	757	HMDB0000115	N.D.	N.D.	N.D.	N.D.	N.D.	N.D.	N.D.	N.D.	N.D.
A_0001	Glyoxylic acid	760	HMDB0000119	N.D.	N.D.	N.D.	N.D.	N.D.	N.D.	N.D.	N.D.	N.D.
A_0129	GMP	6804	HMDB0001397	15	27	37	253	250	314	278	289	117
A_0156	GTP	6830	HMDB0001273	12	11	7.4	2.6	4.4	6.0	6.5	9.0	11
C_0095	Guanine	764	HMDB0000132	1.9	1.5	1.3	2.5	1.9	2.3	3.0	5.6	5.7
C_0192	Guanosine	6802	HMDB0000133	31	31	29	65	53	53	57	71	93
C_0099	His	773	HMDB0000177	782	760	1,081	632	612	546	507	501	499
C_0038	Homoserine	12647	HMDB0000719	1.5	1.6	3.0	2.2	2.2	2.3	1.1	1.1	1.3
C_0059	Hydroxyproline	5810	HMDB0000725	20	19	18	26	22	29	13	12	20
C_0072	Hypoxanthine	790	HMDB0000157	2,383	2,371	2,754	820	709	783	804	998	1,220
C_0063	Ile	791	HMDB0000172	441	433	703	287	289	318	316	325	256
A_0127	IMP	8582	HMDB0000175	11	17	9.8	214	199	222	250	231	64
C_0185	Inosine	6021	HMDB0000195	2,402	2,456	2,179	2,299	2,026	2,020	1,887	2,079	2,554
A_0070	Isocitric acid	1198	HMDB0000193	N.D.	N.D.	N.D.	N.D.	N.D.	N.D.	N.D.	N.D.	N.D.
A_0006	Lactic acid	612	HMDB0000190, HMDB0001311	4,939	5,315	18,093	16,444	15,724	19,917	5,911	4,095	6,285
C_0067	Leu	857	HMDB0000687	802	800	1,421	537	559	613	559	576	525
C_0087	Lys	866	HMDB0000182, HMDB0003405	1,475	1,443	2,295	1,083	1,054	1,264	1,123	874	994
A_0024	Malic acid	525	HMDB0000156, HMDB0000744	1,221	1,545	2,656	2,041	1,917	2,055	450	604	646
A_0142	Malonyl CoA_divalent	644066		N.D.	N.D.	N.D.	N.D.	N.D.	N.D.	N.D.	N.D.	N.D.
C_0093	Met	876	HMDB0000696	387	399	632	224	201	227	267	238	227
C_0017	*N*,*N*-Dimethylglycine	673	HMDB0000092	23	23	22	42	27	46	43	42	40
A_0164	NAD^{+}	5893	HMDB0000902	44	35	23	29	29	40	66	71	55
A_0166	NADP^{+}	5886	HMDB0000217	17	14	8.3	20	16	14	30	32	21
C_0066	Ornithine	389	HMDB0000214, HMDB0003374	669	656	1,073	295	379	402	384	497	554
C_0110	Phe	994	HMDB0000159	387	419	820	298	283	326	311	306	298
A_0044	Phosphoenolpyruvic acid	1005	HMDB0000263	38	41	28	8.5	11	15	13	26	21
C_0032	Pro	614	HMDB0000162, HMDB0003411	597	605	1,076	298	267	338	306	320	312
A_0133	PRPP	7339	HMDB0000280	N.D.	N.D.	N.D.	N.D.	N.D.	N.D.	N.D.	N.D.	N.D.
C_0009	Putrescine	1045	HMDB0001414	3.0	2.2	2.7	4.0	5.1	4.4	5.3	5.7	3.8
A_0004	Pyruvic acid	1060	HMDB0000243	134	N.D.	N.D.	N.D.	113	N.D.	N.D.	129	N.D.
A_0091	Ribose 5-phosphate	439167	HMDB0001548	11	21	46	185	134	113	79	96	68
A_0092	Ribulose 5-phosphate	439184	HMDB0000618	411	382	308	750	672	695	579	630	634
C_0214	*S*-Adenosylmethionine	34755	HMDB0001185	14	9.9	21	38	39	45	23	27	27
C_0011	Sarcosine	1088	HMDB0000271	31	32	59	33	22	34	54	30	34
A_0108	Sedoheptulose 7-phosphate	165007	HMDB0001068	544	331	249	282	90	84	182	225	221
C_0024	Ser	617	HMDB0000187, HMDB0003406	754	836	1,749	633	675	615	541	532	598
C_0085	Spermidine	1102	HMDB0001257	17	17	14	44	46	39	37	35	42
C_0144	Spermine	1103	HMDB0001256	4.8	5.2	5.8	14	14	10	15	19	17
A_0014	Succinic acid	1110	HMDB0000254	582	802	715	538	760	760	469	623	485
C_0039	Thr	6288	HMDB0000167	690	678	1,241	531	503	511	481	452	471
C_0167	Thymidine	5789	HMDB0000273	1.2	N.D.	N.D.	1.5	1.2	1.2	N.D.	N.D.	N.D.
C_0054	Thymine	1135	HMDB0000262	N.D.	N.D.	N.D.	N.D.	N.D.	N.D.	N.D.	N.D.	N.D.
C_0147	Trp	1148	HMDB0000929	71	77	111	65	64	71	59	62	54
C_0128	Tyr	1153	HMDB0000158	378	371	658	266	233	301	262	246	222
C_0076	Tyramine	5610	HMDB0000306	0.4	0.4	0.2	N.D.	0.2	N.D.	0.2	N.D.	0.3
A_0137	UDP	6031	HMDB0000295	4.4	5.3	6.2	6.2	6.0	7.9	6.2	7.5	6.1
A_0119	UMP	6030	HMDB0000288	19	32	42	134	115	155	163	146	52
C_0029	Uracil	1174	HMDB0000300	182	190	228	63	64	68	58	71	96
C_0169	Uridine	6029	HMDB0000296	454	507	762	365	302	314	173	199	253
A_0152	UTP	6133	HMDB0000285	3.7	3.0	2.8	1.0	1.6	N.D.	N.D.	2.4	1.6
C_0034	Val	1182	HMDB0000883	763	766	1,137	468	501	525	523	555	461
C_0010	β-Ala	239	HMDB0000056	441	362	237	125	182	207	294	276	298
