# Supplementary material for: Efficacy of Human Recombinant Growth Hormone in Females of a Non-Obese Hyperglycemic Mouse Model after Birth with Low Birth Weight
Source: Int J Mol Sci. 2024 Jun 7;25(12):6294. doi: 10.3390/ijms25126294 (PMC11203808; doi:10.3390/ijms25126294)
Supplement: Supplementary file 1 [file ijms-25-06294-s001.zip › Supplementary Table S4, PC of muscle.pdf]

Supplementary Table S4. Principle component score of muscle

contribution rate(%)		Control			Ischemia			Ischemia-GH		
		control-C3	control-C4	control-C7	ischemia-I2	ischemia-I4	ischemia-I7	ische-GH-j	ische-GH-k	ische-GH-g
PC1	25.23	-9.95	-3.56	-10.56	-0.71	-0.74	-0.47	0.96	9.66	15.38
PC2	19.37	-1.50	-1.54	10.13	3.52	-11.02	-10.75	6.82	-0.44	4.79
PC3	15.00	14.40	-3.52	-3.59	-4.70	-4.11	-0.84	-2.77	-1.73	6.86
PC4	10.38	-1.78	-3.68	4.56	-1.79	9.54	-3.78	-2.08	-6.72	5.74
PC5	8.62	-2.15	1.94	3.62	3.92	-4.49	5.11	-9.37	-2.28	3.68
PC6	8.33	0.68	10.40	-2.64	-0.24	1.33	-7.28	-3.42	0.23	0.93
PC7	7.75	0.87	-2.78	4.91	-3.26	1.60	-2.66	-5.53	9.07	-2.21
PC8	5.32	1.92	-3.55	-2.83	8.62	1.63	-3.13	-1.88	0.66	-1.43
