# Supplementary material for: Efficacy of Human Recombinant Growth Hormone in Females of a Non-Obese Hyperglycemic Mouse Model after Birth with Low Birth Weight
Source: Int J Mol Sci. 2024 Jun 7;25(12):6294. doi: 10.3390/ijms25126294 (PMC11203808; doi:10.3390/ijms25126294)
Supplement: Supplementary file 1 [file ijms-25-06294-s001.zip › Supplementary Table S5, PC score of muscle.pdf]

Supplementary Table S5. Metabolites and principle component score of muscle

ID	HMT DB † Compound name	PubChem CID	HMDB ID	*m/z*	MT/RT	PC1	PC2
A_0003	Pyruvic acid	1060	HMDB0000243	87.009	10.43	-7.0E-01	-3.3E-01
A_0004	Lactic acid	612	HMDB0000190, HMDB0001311	89.024	9.05	-9.3E-01	1.3E-01
A_0005	Succinic semialdehyde	1112	HMDB0001259	101.024	8.36	-4.8E-01	5.2E-01
A_0006	3-Hydroxybutyric acid	441	HMDB0000011, HMDB0000357, HMDB0000442	103.040	8.05	7.4E-01	1.9E-01
A_0008	Glyceric acid	439194	HMDB0000139, HMDB0006372	105.020	8.67	-4.5E-01	-7.7E-02
A_0009	Fumaric acid	444972	HMDB0000134	115.004	17.24	3.5E-01	3.4E-01
A_0010	2-Oxoisovaleric acid	49	HMDB0000019	115.040	8.52	-1.6E-01	-7.9E-02
A_0011	Succinic acid	1110	HMDB0000254	117.019	15.29	6.9E-01	-2.1E-01
A_0012	β-Hydroxyisovaleric acid-1<br>2-Hydroxyvaleric acid-1<br>2-Hydroxyisovaleric acid-1	69362<br>98009<br>99823	HMDB0000754<br>HMDB0001863<br>HMDB0000407	117.055	7.72	-3.3E-02	-5.7E-01
A_0013	β-Hydroxyisovaleric acid-2<br>2-Hydroxyvaleric acid-2<br>2-Hydroxyisovaleric acid-2	69362<br>98009<br>99823	HMDB0000754<br>HMDB0001863<br>HMDB0000407	117.055	7.83	1.3E-01	9.3E-02
A_0014	Isethionic acid	7866	HMDB0003903	124.991	9.52	-4.9E-02	6.5E-01
A_0015	Monoethyl phosphate	74190	HMDB0012228	125.001	10.88	-5.1E-01	3.1E-01
A_0016	5-Oxoproline	7405	HMDB0000267	128.035	7.99	-5.6E-01	3.5E-01
A_0017	4-Methyl-2-oxovaleric acid<br>3-Methyl-2-oxovaleric acid<br>2-Oxohexanoic acid	70<br>47<br>159664	HMDB0000695<br>HMDB0000491<br>HMDB0001864	129.056	8.05	-4.1E-01	-1.0E-01
A_0018	Malic acid	525	HMDB0000156, HMDB0000744	133.014	15.51	-8.3E-02	-1.4E-01
A_0019	*p*-Toluic acid<br>*o*-Toluic acid<br>*m*-Toluic acid	7470<br>8373<br>7418		135.045	7.73	2.0E-01	-2.5E-01
A_0020	Ethanolamine phosphate	1015	HMDB0000224	140.012	6.82	3.3E-01	-3.0E-01
A_0022	Monomethyl glutaric acid	73917	HMDB0000858	145.050	7.47	-7.4E-01	4.1E-01
A_0023	2-Hydroxyglutaric acid<br>Citramalic acid	43<br>1081	HMDB0000606, HMDB0000694<br>HMDB0000426	147.030	13.17	3.3E-01	-3.6E-01
A_0024	8-Hydroxyoctanoic acid<br>3-Hydroxyoctanoic acid	69820<br>26613	 HMDB0010722	159.101	6.86	-1.1E-01	-5.4E-01
A_0025	Terephthalic acid	7489	HMDB0002428	165.019	12.90	4.8E-02	-4.7E-01
A_0026	*N*-Acetyltaurine	159864	HMDB0240253	166.019	7.92	-5.3E-01	2.7E-01
A_0027	Phosphoenolpyruvic acid	1005	HMDB0000263	166.975	15.43	-1.5E-01	-8.8E-01
A_0028	Uric acid	1175	HMDB0000289	167.021	7.50	-1.6E-01	-8.3E-01
A_0029	2-Amino-3-phosphonopropionic acid	3857	HMDB0000370	168.007	10.36	1.1E-01	3.4E-01
A_0030	Glyceraldehyde 3-phosphate	729	HMDB0001112	168.989	9.68	-4.5E-01	-7.7E-02
A_0031	Dihydroxyacetone phosphate	668	HMDB0001473	168.991	10.40	-4.5E-01	-6.4E-01
A_0032	Glycerol 3-phosphate	439162	HMDB0000126	171.007	9.98	4.4E-01	-6.7E-01
A_0033	Isovalerylalanine<br>*N*-Acetylleucine<br>*N*-Acetylisoleucine<br>*N*-Hexanoylglycine	129285<br>70912<br>7036275<br>99463	HMDB0000747<br>HMDB0011756<br>HMDB0061684<br>HMDB0000701	172.098	6.90	2.5E-01	1.0E-01
A_0034	*cis*-Aconitic acid	643757	HMDB0000072	173.009	18.23	1.9E-02	6.6E-01
A_0035	*N*-Acetylaspartic acid	65065	HMDB0000812	174.041	11.67	-6.4E-01	1.9E-01
A_0036	Ascorbic acid	54670067	HMDB0000044	175.025	7.19	1.2E-01	5.6E-01

A_0037	Hippuric acid	464	HMDB0000714	178.052	7.15	-3.2E-02	9.3E-02
A_0038	3,4-Dihydroxyhydrocinnamic acid<br>Homovanillic acid<br>Hydroxyphenyllactic acid	348154<br>1738<br>9378	HMDB0000423<br>HMDB0000118<br>HMDB0000755	181.051	7.02	-9.5E-01	-1.1E-01
A_0039	3-Phosphoglyceric acid	439183	HMDB0000807	184.986	14.69	-1.4E-01	-8.7E-01
A_0040	2-Phosphoglyceric acid	439278	HMDB0003391	184.986	14.41	-1.6E-01	-8.7E-01
A_0041	Azelaic acid	2266	HMDB0000784	187.098	10.03	1.9E-01	-4.4E-01
A_0042	*N*-Acetylmethionine	448580	HMDB0011745	190.053	6.91	-4.3E-01	4.0E-01
A_0043	Isocitric acid	1198	HMDB0000193	191.019	18.55	-2.8E-01	6.4E-01
A_0044	Citric acid	311	HMDB0000094	191.020	17.66	-2.6E-01	8.7E-01
A_0045	Gluconic acid	10690	HMDB0000625	195.051	6.86	-4.5E-01	5.3E-01
A_0047	Lauric acid	3893	HMDB0000638	199.170	6.55	7.3E-01	3.8E-01
A_0048	Indole-3-lactic acid<br>5-Methoxyindoleacetic acid	676157<br>18986	HMDB0000671<br>HMDB0004096	204.065	6.98	3.1E-02	1.5E-01
A_0049	Phosphocreatine	9548602	HMDB0001511	210.029	10.08	-4.4E-01	-7.1E-01
A_0050	3-Indoxylsulfuric acid	10258	HMDB0000682	212.002	8.04	-3.8E-01	-4.6E-01
A_0051	Pantothenic acid	6613	HMDB0000210	218.103	6.54	-4.5E-01	3.8E-01
A_0052	Myristoleic acid	5281119	HMDB0002000	225.188	6.40	8.6E-01	2.2E-01
A_0053	Ribose 5-phosphate	439167	HMDB0001548	229.011	8.82	-6.6E-01	2.9E-01
A_0054	Ribulose 5-phosphate	439184	HMDB0000618	229.012	9.15	-5.8E-01	4.8E-01
A_0055	7-Amino-4-hydroxy-2-naphthalenesulfonic acid	6868	HMDB0243485	238.017	7.56	6.1E-01	7.9E-02
A_0056	Glucose 1-phosphate	65533	HMDB0001586	259.022	8.49	-5.0E-01	-3.9E-01
A_0057	Glucose 6-phosphate	5958	HMDB0001401	259.022	8.28	-4.5E-01	-5.8E-01
A_0058	Fructose 6-phosphate	603	HMDB0000124	259.023	8.38	-4.5E-01	-5.9E-01
A_0059	*myo*-Inositol 3-phosphate<br>*myo*-Inositol 1-phosphate	440194<br>107737	HMDB0006814<br>HMDB0000213	259.023	8.62	8.4E-01	6.7E-02
A_0060	*myo*-Inositol 2-phosphate	160886		259.023	8.82	-2.3E-01	-7.1E-01
A_0061	Sorbitol 6-phosphate	152306	HMDB0005831	261.037	8.36	-5.6E-01	-6.1E-01
A_0062	2,3-Diphosphoglyceric acid	186004	HMDB0001294	264.949	14.09	-1.0E-01	-8.7E-01
A_0063	6-Phosphogluconic acid	91493	HMDB0001316	275.018	11.78	-2.1E-01	-8.2E-01
A_0064	Sedoheptulose 7-phosphate	165007	HMDB0001068	289.033	8.10	-5.7E-01	5.5E-01
A_0065	*N*-Acetylglucosamine 6-phosphate	440996	HMDB0001062	300.048	7.74	4.1E-01	2.8E-01
A_0066	*N*-Acetylglucosamine 1-phosphate	440272	HMDB0001367	300.049	8.07	-4.5E-02	2.3E-01
A_0067	*N*-Acetylaspartylglutamic acid	5255	HMDB0001067	303.083	12.28	-3.8E-01	4.8E-01
A_0068	*N*-Acetylneuraminic acid	439197	HMDB0000230	308.098	6.17	-7.1E-01	4.2E-02
A_0069	Ribulose 1,5-diphosphate	123658		308.978	12.40	8.8E-02	3.0E-01
A_0071	CMP	6131	HMDB0000095	322.044	8.07	-3.2E-01	5.8E-01
A_0072	UMP	6030	HMDB0000288	323.029	8.20	-5.6E-01	5.3E-01
A_0073	*N*-Glycolylneuraminic acid	440001	HMDB0000833	324.094	6.15	-6.7E-01	6.2E-02
A_0075	Fructose 1,6-diphosphate	172313	HMDB0001058	338.989	11.70	-4.7E-01	-4.2E-01
A_0077	AMP	6083	HMDB0000045	346.055	7.80	5.1E-01	6.8E-01
A_0078	IMP	8582	HMDB0000175	347.039	8.03	-7.7E-01	4.9E-01
A_0079	Maltobionic acid	3036723	HMDB0253945	357.105	6.10	-6.1E-01	-1.2E-01
A_0080	GMP	6804	HMDB0001397	362.051	7.70	-3.1E-01	8.0E-01
A_0081	CoA_divalent	87642	HMDB0001423	382.548	8.86	3.2E-01	5.9E-01
A_0082	PRPP	7339	HMDB0000280	388.943	12.99	-6.2E-01	-1.1E-01
A_0083	FAD_divalent	643975	HMDB0001248	391.571	6.70	3.7E-01	3.7E-01
A_0085	CDP	6132	HMDB0001546	402.013	9.46	-3.5E-01	7.3E-01
A_0086	UDP	6031	HMDB0000295	402.993	9.59	4.9E-01	7.3E-01
A_0087	Acetyl CoA_divalent	444493	HMDB0001206	403.555	8.59	4.4E-01	2.0E-01
A_0089	3',5'-ADP	159296	HMDB0000061	426.022	10.82	8.3E-01	1.6E-01
A_0090	ADP	6022	HMDB0001341	426.022	9.07	5.2E-01	7.8E-01
A_0091	GDP	8977	HMDB0001201	442.016	8.87	6.1E-01	7.2E-01
A_0092	Adenylosuccinic acid	447145	HMDB0000536	462.067	11.12	6.4E-01	3.6E-01
A_0095	CTP	6176	HMDB0000082	481.976	10.12	-8.9E-02	-8.9E-01

A_0096	UTP	6133	HMDB0000285	482.961	10.26	-1.9E-01	-8.6E-01
A_0097	CDP-choline	13804	HMDB0001413	487.099	5.83	-2.1E-01	5.6E-01
A_0099	ATP	5957	HMDB0000538	505.989	9.68	-2.3E-01	-6.7E-01
A_0100	Taurocholic acid	6675	HMDB0000036	514.287	5.78	-4.3E-01	4.2E-01
A_0101	GTP	6830	HMDB0001273	521.983	9.46	-1.4E-01	-8.8E-01
A_0102	ADP-ribose	445794	HMDB0001178	558.065	7.20	-1.6E-01	-2.6E-01
A_0103	UDP-galactose<br>UDP-glucose	23724458<br>8629	HMDB0000302<br>HMDB0000286	565.048	7.30	-9.2E-02	6.3E-01
A_0104	UDP-glucuronic acid	17473	HMDB0000935	579.029	9.17	-7.4E-01	-1.7E-01
A_0105	GDP-fucose<br>ADP-glucose	10918995<br>16500	HMDB0001095<br>HMDB0006557	588.078	7.06	-2.0E-01	6.5E-01
A_0106	GDP-mannose<br>GDP-glucose	18396<br>46173703	HMDB0001163<br>HMDB0003351	604.068	7.03	-5.0E-01	-2.0E-01
A_0107	UDP-*N*-acetylglucosamine<br>UDP-*N*-acetylgalactosamine	445675<br>23724461	HMDB0000290<br>HMDB0000304	606.074	7.15	-1.1E-01	-5.4E-03
A_0108	CMP-N-acetylneuraminate-2	448209	HMDB0001176	613.138	7.04	1.2E-01	2.7E-01
A_0109	CMP-N-acetylneuraminate-1	448209	HMDB0001176	613.138	6.95	-1.5E-01	4.9E-01
A_0110	NAD^+	5893	HMDB0000902	662.102	5.63	7.7E-01	3.9E-01
A_0111	NADH	439153	HMDB0001487	664.114	6.94	1.5E-01	-8.2E-02
A_0112	$NADP^+$	5886	HMDB0000217	742.070	7.89	6.7E-01	-1.8E-01
C_0001	Trimethylamine	1146	HMDB0000906	60.081	4.72	4.4E-01	-3.0E-01
C_0002	Urea	1176	HMDB0000294	61.040	16.26	4.0E-01	3.2E-01
C_0003	Ethanolamine	700	HMDB0000149	62.060	5.09	6.2E-01	5.1E-01
C_0004	Gly	750	HMDB0000123	76.039	6.64	-6.3E-01	1.4E-01
C_0005	Trimethylamine *N*-oxide	1145	HMDB0000925	76.075	5.29	-7.5E-01	-2.2E-02
C_0006	Morpholine	8083	HMDB0031581	88.076	5.32	-9.9E-02	-2.5E-01
C_0007	Putrescine	1045	HMDB0001414	89.107	3.80	-3.0E-01	4.2E-01
C_0008	Sarcosine	1088	HMDB0000271	90.055	7.53	-5.3E-01	-6.3E-01
C_0009	Ala	602	HMDB0000161,<br>HMDB0001310	90.055	7.16	-7.8E-01	7.0E-02
C_0010	β-Ala	239	HMDB0000056	90.055	5.86	6.5E-01	2.8E-01
C_0011	2-Amino-2-methyl-1-propanol	11807	HMDB0244974	90.091	6.15	-7.0E-01	2.1E-01
C_0012	Dimethylaminoethanol	7902	HMDB0032231	90.091	5.53	4.7E-02	1.5E-01
C_0013	Oxalic acid	971	HMDB0002329	91.003	4.19	-6.8E-01	-5.6E-02
C_0014	Glycerol	753	HMDB0000131	93.054	16.91	-1.7E-01	-2.0E-01
C_0015	Cyclohexylamine	7965		100.112	6.09	-1.2E-01	3.2E-01
C_0016	*N*,*N*-Dimethylglycine	673	HMDB0000092	104.070	8.64	-4.0E-02	-2.7E-01
C_0017	2-Aminoisobutyric acid<br>2-Aminobutyric acid	6119<br>6657	HMDB0001906<br>HMDB0000452	104.070	7.62	-6.0E-01	5.1E-01
C_0018	GABA	119	HMDB0000112	104.071	6.13	-3.8E-01	-1.2E-01
C_0019	Choline	305	HMDB0000097	104.107	5.47	3.9E-01	5.4E-01
C_0020	Ser	617	HMDB0000187,<br>HMDB0003406	106.050	7.93	-6.5E-01	-2.4E-01
C_0021	Diethanolamine	8113	HMDB0004437	106.086	6.09	2.6E-02	-2.1E-01
C_0022	Hypotaurine	107812	HMDB0000965	110.027	13.83	4.8E-01	3.1E-01
C_0023	Cytosine	597	HMDB0000630	112.050	5.79	-3.6E-01	7.0E-02
C_0024	Histamine	774	HMDB0000870	112.087	3.86	-1.8E-01	-7.1E-01
C_0025	Uracil	1174	HMDB0000300	113.034	16.91	1.3E-01	-6.4E-01
C_0026	Creatinine	588	HMDB0000562	114.066	5.82	-9.6E-01	6.1E-02
C_0027	3-Amino-2-piperidone	5200225	HMDB0000323	115.086	6.06	-1.3E-01	2.5E-01
C_0028	Pro	614	HMDB0000162,<br>HMDB0003411	116.070	8.52	1.1E-01	4.6E-01
C_0029	Guanidoacetic acid	763	HMDB0000128	118.061	6.55	-1.5E-01	-1.3E-01
C_0030	Betaine	247	HMDB0000043	118.086	8.88	2.3E-01	-1.9E-01

C_0031	Val	1182	HMDB0000883	118.086	7.90 1.9E-01	7.8E-01
C_0032	Homoserine	12647	HMDB0000719	120.065	7.98 -3.5E-01	-3.5E-02
C_0033	Thr	6288	HMDB0000167	120.065	8.32 -5.4E-01	-2.8E-01
C_0034	Betaine aldehyde_+H_2O	249	HMDB0001252	120.101	5.94 -4.0E-02	-7.3E-01
C_0035	Anserine_divalent	112072	HMDB0000194	121.068	5.44 -7.5E-01	1.3E-01
C_0036	Cys	594	HMDB0000574, HMDB0003417	122.027	8.92 -2.5E-01	7.8E-01
C_0037	Isonicotinamide<br>Nicotinamide	15074<br>936	HMDB0001406	123.055	5.92 -6.9E-01	4.3E-01
C_0038	Taurine	1123	HMDB0000251	126.022	16.91 -2.6E-01	8.2E-01
C_0039	1-Methylhistamine	3614	HMDB0000898	126.102	3.97 -6.8E-01	-2.2E-01
C_0041	Imidazole-4-acetic acid	96215	HMDB0002024	127.050	6.39 1.0E-01	-4.4E-01
C_0042	5-Amino-3,4-dihydro-2H-pyrrole-2-carboxylic acid	13894665		129.065	6.88 -2.3E-01	7.8E-01
C_0043	4-Oxopyrrolidine-2-carboxylic acid	107541		130.050	8.65 3.1E-01	6.8E-01
C_0044	Pipecolic acid	439227	HMDB0000070, HMDB0000716, HMDB0005960	130.086	8.10 -5.8E-01	3.5E-01
C_0045	*trans*-Glutaconic acid	5280498	HMDB0000620	131.033	17.49 -7.8E-02	-8.9E-02
C_0046	Hydroxyproline	5810	HMDB0000725	132.065	9.45 2.0E-01	-1.8E-01
C_0047	Ile	791	HMDB0000172	132.101	8.04 4.0E-01	6.8E-01
C_0048	Leu	857	HMDB0000687	132.101	8.13 2.7E-01	6.6E-01
C_0049	6-Aminohexanoic acid	564	HMDB0001901	132.102	6.60 6.9E-01	2.5E-01
C_0050	Gly-Gly	11163	HMDB0011733	133.060	6.64 -4.2E-02	7.4E-01
C_0051	Asn	236	HMDB0000168, HMDB0033780	133.060	8.32 -5.0E-01	3.1E-01
C_0052	Creatine	586	HMDB0000064	133.079	6.89 -5.7E-01	5.3E-01
C_0053	Ornithine	389	HMDB0000214, HMDB0003374	133.097	5.44 -4.8E-01	-4.3E-01
C_0054	Thiaproline	9934		134.027	10.93 1.3E-01	5.5E-01
C_0055	Asp	424	HMDB0000191, HMDB0006483	134.044	9.14 1.4E-01	8.9E-01
C_0056	Adenine	190	HMDB0000034	136.061	6.08 5.1E-01	2.1E-01
C_0057	Hypoxanthine	790	HMDB0000157	137.046	8.96 -1.2E-01	5.1E-01
C_0058	1-Methylnicotinamide	457	HMDB0000699	137.071	5.87 -5.4E-01	-1.2E-01
C_0060	Trigonelline	5570	HMDB0000875	138.055	8.27 -8.2E-01	-1.5E-01
C_0061	Tyramine	5610	HMDB0000306	138.091	6.60 5.8E-02	5.6E-01
C_0062	γ-Glu-Lys_divalent	65254	HMDB0029154	138.580	6.79 -3.7E-01	-8.6E-01
C_0063	Urocanic acid	736715	HMDB0000301	139.050	6.59 -5.7E-01	2.5E-01
C_0064	1-Methyl-4-imidazoleacetic acid-2	75810	HMDB0002820	141.065	6.59 -5.5E-01	3.5E-01
C_0065	1*H*-Imidazole-4-propionic acid	10105257		141.065	6.44 -5.2E-01	-1.8E-01
C_0066	1-Methyl-4-imidazoleacetic acid-1	75810	HMDB0002820	141.065	6.55 -3.4E-01	-2.3E-02
C_0067	Ectoine	126041		143.081	7.44 -5.3E-01	4.4E-01
C_0068	Stachydrine	115244	HMDB0004827	144.101	9.02 -7.1E-01	-2.4E-02
C_0069	Crotonic acid betaine	5280649	HMDB0250545	144.102	6.57 -4.5E-01	-7.7E-02
C_0070	4-Guanidinobutyric acid	500	HMDB0003464	146.092	6.58 -8.0E-01	1.7E-02
C_0071	γ-Butyrobetaine	134	HMDB0001161	146.117	6.44 -8.5E-01	4.3E-01
C_0072	Spermidine	1102	HMDB0001257	146.165	3.66 -7.1E-01	-1.2E-01
C_0073	Gln	738	HMDB0000641, HMDB0003423	147.076	8.49 -3.4E-01	4.3E-01
C_0074	Glycylsarcosine	93131	HMDB0252887	147.077	6.84 -4.5E-01	-7.7E-02
C_0075	Lys	866	HMDB0000182, HMDB0003405	147.112	5.49 -9.4E-01	-1.8E-02
C_0076	2-Methylthiazolidine-4-carboxylic acid	160736	HMDB0246657	148.042	11.10 3.3E-01	7.0E-01

C_0077	Glu	611	HMDB0000148,<br>HMDB0003339	148.060	8.65	2.9E-01	7.4E-01
C_0078	Met	876	HMDB0000696	150.058	8.46	-9.1E-01	1.2E-01
C_0080	Xanthine	1188	HMDB0000292	153.040	15.12	7.8E-02	-4.5E-01
C_0081	N^1-Methyl-4-pyridone-5-carboxamide	440810	HMDB0004194	153.065	14.29	-8.2E-01	-1.2E-02
C_0082	His	773	HMDB0000177	156.076	5.84	-4.2E-01	7.2E-01
C_0083	Tranexamic acid	5526	HMDB0014447	158.117	6.87	6.6E-01	-1.8E-01
C_0084	Ala-Ala	5460362	HMDB0003459	161.091	7.29	-8.1E-01	2.1E-02
C_0085	N^6-Methyllysine	164795	HMDB0002038	161.128	5.66	-8.8E-01	1.9E-01
C_0086	Carnitine	85	HMDB0000062	162.112	6.75	-3.3E-02	7.2E-01
C_0087	5-Hydroxylysine	3032849	HMDB0000450	163.107	5.71	-7.6E-01	-6.8E-02
C_0088	Methionine sulfoxide	158980	HMDB0002005	166.053	9.28	-8.0E-01	1.6E-01
C_0089	Phe	994	HMDB0000159	166.086	8.73	-1.7E-01	6.2E-01
C_0090	Taurocyamine	68340	HMDB0003584	168.043	16.93	-1.0E-01	8.0E-01
C_0091	1-Methylhistidine<br>3-Methylhistidine	92105<br>64969	HMDB0000001<br>HMDB0000479	170.092	5.98	-2.2E-01	3.2E-01
C_0092	N^5-Ethylglutamine	439378	HMDB0034365	175.107	8.94	-6.0E-01	3.1E-01
C_0093	*N*-Acetylornithine	439232	HMDB0003357	175.108	7.51	-5.6E-01	7.7E-02
C_0094	Arg	6322	HMDB0000517,<br>HMDB0003416	175.119	5.68	-9.1E-01	9.6E-02
C_0095	Citrulline	9750	HMDB0000904	176.103	8.72	-8.7E-01	1.0E-01
C_0096	Serotonin	5202	HMDB0000259	177.103	6.87	-3.2E-01	-3.5E-01
C_0097	Gluconolactone	7027	HMDB0000150	179.054	17.52	5.6E-01	-1.5E-01
C_0098	Galactosamine<br>Glucosamine	24154<br>439213	HMDB0001514	180.086	7.30	1.6E-01	2.9E-01
C_0099	Tyr	1153	HMDB0000158	182.081	8.93	-8.0E-01	4.3E-01
C_0100	Phosphorylcholine	1014	HMDB0001565	184.073	15.69	-8.0E-01	-5.9E-02
C_0101	N^1-Acetylspermidine	496	HMDB0001276	188.176	5.06	-1.9E-01	-1.9E-02
C_0102	*N*-Acetyllysine	92907	HMDB0000446	189.123	7.71	-7.5E-01	8.0E-02
C_0103	N^6-Acetyllysine	92832	HMDB0000206	189.123	8.99	-6.0E-01	-2.8E-01
C_0104	N^{ω}-Methylarginine	132862		189.134	5.94	-9.4E-01	-1.8E-01
C_0105	N^6,N^6,N^6-Trimethyllysine	440120	HMDB0001325	189.159	5.73	-8.0E-01	3.7E-01
C_0106	Gly-Gly-Gly	11161	HMDB0029419	190.083	7.43	5.1E-01	1.8E-01
C_0107	Homocitrulline	65072	HMDB0000679	190.118	8.80	-5.5E-02	6.6E-01
C_0108	H-Asp(Gly-OH)-OH	99717	HMDB0011165	191.064	9.42	-2.6E-01	-5.5E-01
C_0109	Gly-Asp	97363		191.066	7.81	-8.6E-02	8.1E-01
C_0110	γ-Carboxyglutamic acid	40772	HMDB0041900	192.049	11.29	-2.2E-01	3.5E-01
C_0111	11-Aminoundecanoic acid	17083		202.180	7.59	-7.9E-01	1.7E-01
C_0112	ADMA	123831	HMDB0001539	203.148	6.10	-8.5E-01	2.2E-01
C_0113	SDMA	169148	HMDB0003334	203.150	6.20	-8.2E-01	3.9E-01
C_0114	Spermine	1103	HMDB0001256	203.223	3.61	-6.2E-01	-3.8E-01
C_0115	*O*-Acetylcarnitine	439756	HMDB0000201	204.123	7.07	-4.3E-01	7.7E-01
C_0116	γ-Glu-Gly	165527	HMDB0011667	205.081	9.46	-2.5E-02	7.9E-01
C_0117	Trp	1148	HMDB0000929	205.097	8.68	-7.8E-01	1.7E-01
C_0118	Carboxymethyllysine	123800		205.118	7.32	-7.7E-02	6.2E-01
C_0119	Kynurenine	846	HMDB0000684	209.091	7.93	-4.9E-01	-8.2E-03
C_0120	Glycerophosphorylethanolamine	444183	HMDB0000114	216.063	16.59	-2.2E-01	2.7E-01
C_0121	Propionylcarnitine	188824	HMDB0000824	218.138	7.34	7.7E-01	1.7E-01
C_0122	β-Ala-Lys	440638		218.150	5.35	-7.0E-01	2.8E-02
C_0123	*N*-Acetylgalactosamine<br>*N*-Acetylglucosamine<br>*N*-Acetylmannosamine	35717<br>439174<br>439281	HMDB0000853<br>HMDB0000215<br>HMDB0001129	222.096	16.94	-5.2E-01	3.2E-02
C_0124	Cystathionine	834	HMDB0000099	223.074	7.85	2.8E-01	5.9E-01
C_0125	3-Nitrotyrosine	65124	HMDB0001904	227.067	9.56	4.3E-01	-2.3E-02
C_0126	Carnosine	439224	HMDB0000033	227.114	5.38	-2.5E-01	1.7E-01

C_0127	2'-Deoxycytidine	13711	HMDB0000014	228.098	7.44	-5.4E-01	-5.0E-02
C_0128	Butyrylcarnitine	439829	HMDB0002013	232.154	7.55	5.7E-01	1.2E-01
C_0129	Thr-Asp	3280446		235.092	8.36	-4.1E-01	8.4E-02
C_0130	γ-Glu-Ser	22844748	HMDB0029158	235.092	9.89	-3.5E-01	-5.0E-02
C_0131	Ser-Glu			235.092	8.19	-7.3E-01	8.7E-02
C_0132	Cystine	595	HMDB0000192	241.030	8.69	6.2E-01	4.6E-01
C_0133	Homocarnosine	10243361	HMDB0000745	241.129	5.44	-7.7E-01	-2.2E-02
C_0134	Thymidine	5789	HMDB0000273	243.097	16.97	-6.2E-01	-5.7E-01
C_0135	Cytidine	6175	HMDB0000089	244.093	7.63	3.1E-01	7.0E-01
C_0136	Uridine	6029	HMDB0000296	245.077	16.96	9.6E-03	4.2E-01
C_0137	Isovalerylcarnitine	6426851	HMDB0000688	246.170	7.70	-7.8E-01	1.3E-01
C_0138	γ-Glu-Val	7015683	HMDB0011172	247.130	9.95	-4.6E-01	-1.2E-01
C_0139	Malonylcarnitine	22833583	HMDB0002095	248.112	8.05	-3.9E-01	5.9E-01
C_0140	Pyridoxamine 5'-phosphate	1053	HMDB0001555	249.063	8.26	-2.1E-01	7.6E-01
C_0141	γ-Glu-Thr	53861142	HMDB0029159	249.107	9.97	6.9E-01	2.5E-01
C_0142	γ-Glu-Cys	123938	HMDB0001049	251.069	10.03	3.6E-01	7.2E-01
C_0143	Nicotinamide riboside	439924	HMDB0000855	255.101	7.49	3.6E-01	-4.9E-01
C_0144	2'-*O*-Methylcytidine	150971	HMDB0242132	258.109	7.79	-3.3E-02	-5.7E-01
C_0145	Glycerophosphocholine	439285	HMDB0000086	258.110	16.58	1.3E-03	-1.2E-01
C_0146	γ-Glu-Ile<br>γ-Glu-Leu	22885096<br>151023	HMDB0011170<br>HMDB0011171	261.143	10.10	5.6E-01	1.4E-01
C_0147	Succinylcarnitine	71464481	HMDB0061717	262.128	7.82	2.2E-01	7.8E-01
C_0148	γ-Glu-Ornitine<br>H-Asp(Lys-OH)-OH	189156<br>165361	HMDB0002248	262.140	6.71	-5.9E-01	3.0E-01
C_0149	3-Hydroxyisovalerylcarnitine	57357187	HMDB0061189	262.165	7.94	-5.4E-01	6.9E-01
C_0150	γ-Glu-Asp	161197	HMDB0030419	263.087	10.18	-3.3E-01	-1.9E-02
C_0151	Thiamine	1130	HMDB0000235	265.112	5.25	1.2E-01	-3.1E-01
C_0152	2'-Deoxyguanosine	187790	HMDB0000085	268.102	9.14	-2.9E-01	-2.9E-01
C_0153	Adenosine	60961	HMDB0000050	268.104	7.80	7.4E-01	4.1E-01
C_0154	Inosine	6021	HMDB0000195	269.088	15.10	-2.7E-02	2.0E-01
C_0155	N^{α}-Succinylarginine	439968	HMDB0032764	275.134	8.36	-5.0E-01	2.6E-01
C_0156	γ-Glu-Gln	150914	HMDB0011738	276.119	10.18	7.6E-01	1.2E-01
C_0157	γ-Glu-Glu	92865	HMDB0011737	277.102	10.25	6.6E-01	2.4E-02
C_0158	Glu-Glu	439500		277.103	8.59	-6.8E-01	5.5E-02
C_0159	1-Methyladenosine	27476	HMDB0003331	282.120	7.85	-6.5E-01	4.2E-01
C_0160	Guanosine	6802	HMDB0000133	284.098	9.94	4.1E-01	1.3E-01
C_0161	His-Glu	7010583		285.118	6.02	-1.2E-01	8.4E-02
C_0162	N^{4}-Acetylcytidine	107461	HMDB0005923	286.102	13.76	-7.5E-01	8.7E-02
C_0163	Octanoylcarnitine	11953814	HMDB0000791	288.216	8.20	2.8E-01	-6.8E-01
C_0164	Ophthalmic acid	7018721	HMDB0005765	290.134	10.35	-3.9E-01	2.3E-01
C_0165	Argininosuccinic acid	16950	HMDB0000052	291.129	7.47	-8.9E-01	2.2E-01
C_0166	5'-Deoxy-5'-methylthioadenosine	439176	HMDB0001173	298.097	7.96	-1.2E-01	2.3E-01
C_0167	Arg-Glu			304.160	5.97	-6.8E-01	-1.6E-01
C_0168	Glutathione (GSSG)_divalent	65359	HMDB0003337	307.083	9.56	4.8E-02	3.1E-01
C_0169	Glutathione (GSH)	124886	HMDB0000125	308.091	10.36	-8.6E-01	3.2E-01
C_0170	Tyr-Glu			311.123	8.75	-6.8E-01	3.5E-01
C_0171	NMN	14180	HMDB0000229	335.063	15.81	-3.0E-01	7.2E-01
C_0172	Thiamine phosphate	1131	HMDB0002666	345.077	8.41	4.3E-01	3.8E-01
C_0173	Decarboxylated *S*-Adenosylmethionine	439415	HMDB0000988	355.154	4.50	5.0E-01	2.1E-01
C_0174	*S*-Lactoylglutathione	440018	HMDB0001066	380.112	10.78	-4.8E-01	-4.1E-01
C_0175	Succinyladenosine	165243	HMDB0000912	384.112	11.38	7.5E-01	-4.5E-01
C_0176	*S*-Adenosylhomocysteine	439155	HMDB0000939	385.129	6.88	-2.3E-01	-2.2E-01
C_0177	*S*-Adenosylmethionine	34755	HMDB0001185	399.144	5.69	-6.0E-01	3.7E-01
C_0178	Cysteine glutathione disulfide	10455148	HMDB0000656	427.095	9.17	5.0E-01	4.6E-01
C_0179	CDP-ethanolamine	123727	HMDB0001564	447.067	16.51	-1.6E-01	-4.5E-03
