# Supplementary material for: Efficacy of Human Recombinant Growth Hormone in Females of a Non-Obese Hyperglycemic Mouse Model after Birth with Low Birth Weight
Source: Int J Mol Sci. 2024 Jun 7;25(12):6294. doi: 10.3390/ijms25126294 (PMC11203808; doi:10.3390/ijms25126294)
Supplement: Supplementary file 1 [file ijms-25-06294-s001.zip › Supplementary Table S6, metabolites of muscle.pdf]

Supplemantary Table S6. Concentrations of all metabolites of muscle

ID	Metabolite	PubChem CID	HMDB ID	Concentration (nmol/g)								
				Control			Ischemia			Ischemia-GH		
				control-C3	control-C4	control-C7	ischemia-I2	ischemia-I4	ischemia-I7	ische-GH-j	ische-GH-k	ische-GH-g
A_0007	2-Hydroxybutyric acid	440864	HMDB0000008	N.D.	N.D.	N.D.	N.D.	N.D.	N.D.	N.D.	N.D.	N.D.
A_0021	2-Oxoglutaric acid	51	HMDB0000208	N.D.	N.D.	N.D.	N.D.	N.D.	N.D.	N.D.	N.D.	N.D.
A_0010	2-Oxoisovaleric acid	49	HMDB0000019	N.D.	7.7	N.D.	N.D.	N.D.	N.D.	N.D.	N.D.	N.D.
A_0040	2-Phosphoglyceric acid	439278	HMDB0003391	48	N.D.	N.D.	N.D.	120	151	N.D.	N.D.	N.D.
A_0006	3-Hydroxybutyric acid	441	HMDB0000011, HMDB0000357, HMDB0000442	83	127	170	125	146	173	274	513	305
A_0039	3-Phosphoglyceric acid	439183	HMDB0000807	307	36	N.D.	N.D.	960	1,219	N.D.	N.D.	N.D.
A_0063	6-Phosphogluconic acid	91493	HMDB0001316	23	13	12	11	21	27	14	14	13
A_0087	Acetyl CoA_divalent	444493		1.3	N.D.	N.D.	N.D.	N.D.	N.D.	N.D.	N.D.	2.7
C_0056	Adenine	190	HMDB0000034	0.5	N.D.	N.D.	N.D.	N.D.	N.D.	N.D.	N.D.	1.2
C_0153	Adenosine	60961	HMDB0000050	1.0	0.7	1.4	1.5	0.8	0.8	1.0	1.8	4.1
A_0090	ADP	6022	HMDB0001341	11	12	153	182	7.1	9.8	179	102	270
C_0009	Ala	602	HMDB0000161, HMDB0001310	1,844	1,629	1,837	1,575	1,597	1,801	1,818	1,338	1,468
A_0077	AMP	6083	HMDB0000045	1.7	N.D.	86	153	N.D.	2.9	117	29	225
C_0059	Anthranilic acid	227	HMDB0001123	N.D.	N.D.	N.D.	N.D.	N.D.	N.D.	N.D.	N.D.	N.D.
C_0094	Arg	6322	HMDB0000517, HMDB0003416	764	801	1,010	564	769	461	588	298	238
C_0051	Asn	236	HMDB0000168, HMDB0033780	143	110	123	132	74	128	119	107	98
C_0055	Asp	424	HMDB0000191, HMDB0006483	113	102	169	165	51	94	134	131	159
A_0099	ATP	5957	HMDB0000538	483	359	196	156	382	341	220	309	256
C_0030	Betaine	247	HMDB0000043	61	50	57	65	61	63	55	55	65
C_0034	Betaine aldehyde_+H_2O	249		N.D.	N.D.	N.D.	N.D.	2.0	0.6	N.D.	N.D.	N.D.
A_0074	cAMP	6076	HMDB0000058	N.D.	N.D.	N.D.	N.D.	N.D.	N.D.	N.D.	N.D.	N.D.
C_0126	Carnosine	439224	HMDB0000033	771	656	1,149	680	1,190	524	752	492	918
A_0085	CDP	6132	HMDB0001546	N.D.	N.D.	5.4	3.9	N.D.	N.D.	4.9	N.D.	N.D.
A_0076	cGMP	24316	HMDB0001314	N.D.	N.D.	N.D.	N.D.	N.D.	N.D.	N.D.	N.D.	N.D.
C_0019	Choline	305	HMDB0000097	159	80	195	179	159	108	159	195	214
A_0034	*cis*-Aconitic acid	643757	HMDB0000072	10	N.D.	14	12	8.3	N.D.	13	12	9.1
A_0044	Citric acid	311	HMDB0000094	271	261	338	325	187	123	290	253	242
C_0095	Citrulline	9750	HMDB0000904	208	195	251	216	185	195	158	158	134
A_0071	CMP	6131	HMDB0000095	45	52	56	71	29	46	61	44	37
A_0081	CoA_divalent	87642		3.5	2.1	3.6	2.4	2.4	2.1	3.2	3.9	3.9
C_0052	Creatine	586	HMDB0000064	18,568	15,596	16,265	14,974	12,567	13,399	17,018	12,936	14,372
C_0026	Creatinine	588	HMDB0000562	92	77	93	71	80	66	76	55	46
A_0095	CTP	6176	HMDB0000082	12	11	N.D.	N.D.	19	13	5.7	10	2.4
C_0036	Cys	594	HMDB0000574, HMDB0003417	0.6	1.8	15	10	0.4	0.4	11	5.3	1.4
C_0135	Cytidine	6175	HMDB0000089	12	7.7	12	10	9.5	6.0	12	12	14
C_0023	Cytosine	597	HMDB0000630	N.D.	N.D.	0.5	0.5	0.5	0.4	0.5	N.D.	N.D.
A_0098	dATP	15993	HMDB0001532	N.D.	N.D.	N.D.	N.D.	N.D.	N.D.	N.D.	N.D.	N.D.
A_0093	dCTP	65091	HMDB0000998	N.D.	N.D.	N.D.	N.D.	N.D.	N.D.	N.D.	N.D.	N.D.
A_0031	Dihydroxyacetone phosphate	668	HMDB0001473	1,551	236	N.D.	N.D.	679	1,009	N.D.	40	N.D.
A_0084	dTDP	164628	HMDB0001274	N.D.	N.D.	N.D.	N.D.	N.D.	N.D.	N.D.	N.D.	N.D.
A_0070	dTMP	9700	HMDB0001227	N.D.	N.D.	N.D.	N.D.	N.D.	N.D.	N.D.	N.D.	N.D.
A_0094	dTTP	64968	HMDB0001342	N.D.	N.D.	N.D.	N.D.	N.D.	N.D.	N.D.	N.D.	N.D.
A_0046	Erythrose 4-phosphate	122357	HMDB0001321	N.D.	N.D.	N.D.	N.D.	N.D.	N.D.	N.D.	N.D.	N.D.
A_0075	Fructose 1,6-diphosphate	172313	HMDB0001058	5,054	64	8.1	N.D.	1,387	1,732	11	N.D.	N.D.
A_0058	Fructose 6-phosphate	603	HMDB0000124	1,026	72	72	22	372	782	36	23	17
A_0009	Fumaric acid	444972	HMDB0000134	294	150	208	223	192	234	308	216	331
C_0018	GABA	119	HMDB0000112	6.5	3.9	10	5.6	12	3.8	4.0	4.1	5.1
A_0091	GDP	8977	HMDB0001201	N.D.	N.D.	12	15	N.D.	N.D.	13	11	25
C_0073	Gln	738	HMDB0000641, HMDB0003423	2,787	2,030	2,498	2,446	1,465	2,506	2,359	1,885	2,337
C_0077	Glu	611	HMDB0000148, HMDB0003339	1,250	1,047	1,811	1,341	631	926	1,093	1,242	2,144
A_0045	Gluconic acid	10690	HMDB0000625	N.D.	19	21	N.D.	N.D.	N.D.	14	N.D.	N.D.
A_0056	Glucose 1-phosphate	65533	HMDB0001586	573	55	45	32	134	236	32	28	17
A_0057	Glucose 6-phosphate	5958	HMDB0001401	3,726	256	279	86	1,299	2,840	141	66	36
C_0169	Glutathione (GSH)	124886	HMDB0000125	819	673	794	714	513	439	515	428	387
C_0168	Glutathione (GSSG)_divalent	65359		139	40	59	47	24	22	31	32	131
C_0004	Gly	750	HMDB0000123	1,555	1,541	1,771	1,687	1,434	1,779	1,662	1,466	1,331
A_0030	Glyceraldehyde 3-phosphate	729	HMDB0001112	54	N.D.	N.D.	N.D.	N.D.	N.D.	N.D.	N.D.	N.D.
A_0032	Glycerol 3-phosphate	439162	HMDB0000126	435	253	57	45	880	1,174	480	457	888
A_0002	Glycolic acid	757	HMDB0000115	N.D.	N.D.	N.D.	N.D.	N.D.	N.D.	N.D.	N.D.	N.D.
A_0001	Glyoxylic acid	760	HMDB0000119	N.D.	N.D.	N.D.	N.D.	N.D.	N.D.	N.D.	N.D.	N.D.
A_0080	GMP	6804	HMDB0001397	150	148	164	143	71	115	152	112	140
A_0101	GTP	6830	HMDB0001273	47	37	18	17	67	44	24	34	23
C_0079	Guanine	764	HMDB0000132	N.D.	N.D.	N.D.	N.D.	N.D.	N.D.	N.D.	N.D.	N.D.
C_0160	Guanosine	6802	HMDB0000133	3.6	1.8	2.9	2.0	3.5	1.8	2.8	3.9	4.0
C_0082	His	773	HMDB0000177	236	209	243	214	134	198	243	187	195

C_0032	Homoserine	12647	HMDB0000719	1.4	1.7	2.1	1.9	1.7	2.2	1.7	1.5	1.5
C_0046	Hydroxyproline	5810	HMDB0000725	75	133	127	152	95	184	85	119	134
C_0057	Hypoxanthine	790	HMDB0000157	155	98	161	103	97	102	109	144	135
C_0047	Ile	791	HMDB0000172	99	92	141	120	109	105	176	136	147
A_0078	IMP	8582	HMDB0000175	13,515	12,363	13,342	12,077	10,090	11,507	12,805	10,398	10,588
C_0154	Inosine	6021	HMDB0000195	404	169	282	175	190	208	192	301	307
A_0043	Isocitric acid	1198	HMDB0000193	N.D.	N.D.	6.4	N.D.	N.D.	N.D.	7.9	N.D.	N.D.
A_0004	Lactic acid	612	HMDB0000190, HMDB0001311	76,583	64,641	71,209	60,498	57,337	62,896	64,223	49,693	49,651
C_0048	Leu	857	HMDB0000687	199	156	228	181	181	162	265	241	220
C_0075	Lys	866	HMDB0000182, HMDB0003405	2,457	2,454	2,556	1,644	2,142	1,439	1,683	773	622
A_0018	Malic acid	525	HMDB0000156, HMDB0000744	1,155	660	838	768	840	1,081	1,001	782	973
A_0088	Malonyl CoA_divalent	644066		N.D.	N.D.	N.D.	N.D.	N.D.	N.D.	N.D.	N.D.	N.D.
C_0078	Met	876	HMDB0000696	88	76	96	76	64	81	69	65	52
C_0016	*N*,*N*-Dimethylglycine	673	HMDB0000092	6.5	6.8	7.0	7.9	8.1	7.7	8.2	7.1	6.5
A_0110	NAD^+	5893	HMDB0000902	19	57	73	63	91	38	124	110	147
A_0112	$NADP^+$	5886	HMDB0000217	2.9	N.D.	N.D.	N.D.	6.0	N.D.	N.D.	6.1	7.9
C_0053	Ornithine	389	HMDB0000214, HMDB0003374	25	22	26	27	28	28	20	19	23
C_0089	Phe	994	HMDB0000159	125	98	121	95	86	96	137	119	102
A_0027	Phosphoenolpyruvic acid	1005	HMDB0000263	95	12	N.D.	N.D.	278	348	N.D.	N.D.	N.D.
C_0028	Pro	614	HMDB0000162, HMDB0003411	188	235	294	249	227	257	268	262	241
A_0082	PRPP	7339	HMDB0000280	14	N.D.	14	N.D.	15	N.D.	N.D.	N.D.	N.D.
C_0007	Putrescine	1045	HMDB0001414	7.3	5.4	12	9.1	7.7	7.3	6.3	8.2	7.3
A_0003	Pyruvic acid	1060	HMDB0000243	930	523	532	247	471	631	402	335	260
A_0053	Ribose 5-phosphate	439167	HMDB0001548	113	64	77	67	28	67	71	44	47
A_0054	Ribulose 5-phosphate	439184	HMDB0000618	495	317	407	347	210	299	338	285	309
C_0177	*S*-Adenosylmethionine	34755	HMDB0001185	18	21	24	22	16	21	22	18	14
C_0008	Sarcosine	1088	HMDB0000271	9.7	8.6	5.0	7.6	9.8	6.6	2.9	4.8	3.6
A_0064	Sedoheptulose 7-phosphate	165007	HMDB0001068	8.6	15	23	18	10	10	16	8.8	6.6
C_0020	Ser	617	HMDB0000187, HMDB0003406	533	482	480	489	360	613	403	349	337
C_0072	Spermidine	1102	HMDB0001257	53	29	45	33	40	43	44	34	26
C_0114	Spermine	1103	HMDB0001256	197	50	58	33	87	92	40	18	26
A_0011	Succinic acid	1110	HMDB0000254	185	148	82	107	257	259	321	225	350
C_0033	Thr	6288	HMDB0000167	472	422	461	481	365	592	384	369	339
C_0134	Thymidine	5789	HMDB0000273	2.0	2.7	2.2	N.D.	3.3	2.3	N.D.	N.D.	N.D.
C_0040	Thymine	1135	HMDB0000262	N.D.	N.D.	N.D.	N.D.	N.D.	N.D.	N.D.	N.D.	N.D.
C_0117	Trp	1148	HMDB0000929	46	43	40	34	30	33	38	35	25
C_0099	Tyr	1153	HMDB0000158	184	145	235	190	143	132	141	113	114
C_0061	Tyramine	5610	HMDB0000306	N.D.	0.2	0.3	0.4	0.3	N.D.	0.4	N.D.	0.3
A_0086	UDP	6031	HMDB0000295	N.D.	N.D.	6.9	13	N.D.	N.D.	9.3	6.2	13
A_0072	UMP	6030	HMDB0000288	154	131	145	140	83	128	159	107	106
C_0025	Uracil	1174	HMDB0000300	4.9	4.5	5.9	4.5	7.6	7.6	5.4	6.0	5.5
C_0136	Uridine	6029	HMDB0000296	34	22	29	28	25	21	27	33	28
A_0096	UTP	6133	HMDB0000285	27	18	8.6	9.5	44	23	9.7	20	8.0
C_0031	Val	1182	HMDB0000883	259	240	313	282	241	254	346	300	289
C_0010	β-Ala	239	HMDB0000056	8.1	13	32	26	24	25	19	14	111
